# Reversal of Hyperglycemia by Insulin-Secreting Rat Bone Marrow- and Blastocyst-Derived Hypoblast Stem Cell-Like Cells

**DOI:** 10.1371/journal.pone.0063491

**Published:** 2013-05-09

**Authors:** Anujith Kumar, Antonio Lo Nigro, Conny Gysemans, Qing Cai, Camila Esguerra, Molly Nelson-Holte, Yves Heremans, María Jiménez-González, Angelo Porciuncula, Chantal Mathieu, Bert Binas, Harry Heimberg, Felipe Prosper, Bernhard Hering, Catherine M. Verfaillie, Miguel Barajas

**Affiliations:** 1 Stamcel Instituut Leuven, Katholieke Universiteit Leuven, Leuven, Belgium; 2 Laboratory for Experimental Medicine and Endocrinology, Katholieke Universiteit Leuven, Leuven, Belgium; 3 Stem Cell Institute, University of Minnesota, Minneapolis, Minnesota, United States of America; 4 Diabetes Research Center, Vrije Universiteit Brussel, Brussels, Belgium; 5 Hematology and Cell Therapy, Clinica Universidad de Navarra and Foundation for Applied Medical Research, Division of Oncology, University of Navarra, Pamplona, Spain; 6 Division of Molecular & Life Science, College of Science and Technology, Hanyang University, Ansan, South Korea; 7 Diabetes Institute for Immunology and Transplantation. University of Minnesota, Minneapolis, Minnesota, United States of America; 8 Manipal Institute of Regenerative Medicine, Domlur, Bangalore, India; Children's Hospital Boston, United States of America

## Abstract

β-cell replacement may efficiently cure type 1 diabetic (T1D) patients whose insulin-secreting β-cells have been selectively destroyed by autoantigen-reactive T cells. To generate insulin-secreting cells we used two cell sources: rat multipotent adult progenitor cells (rMAPC) and the highly similar rat extra-embryonic endoderm precursor (rXEN-P) cells isolated under rMAPC conditions from blastocysts (rHypoSC). rMAPC/rHypoSC were sequentially committed to definitive endoderm, pancreatic endoderm, and β-cell like cells. On day 21, 20% of rMAPC/rHypoSC progeny expressed Pdx1 and C-peptide. rMAPCr/HypoSC progeny secreted C-peptide under the stimulus of insulin agonist carbachol, and was inhibited by the L-type voltage-dependent calcium channel blocker nifedipine. When rMAPC or rHypoSC differentiated d21 progeny were grafted under the kidney capsule of streptozotocin-induced diabetic nude mice, hyperglycemia reversed after 4 weeks in 6/10 rMAPC- and 5/10 rHypoSC-transplanted mice. Hyperglycemia recurred within 24 hours of graft removal and the histological analysis of the retrieved grafts revealed presence of Pdx1-, Nkx6.1- and C-peptide-positive cells. The ability of both rMAPC and HypoSC to differentiate to functional β-cell like cells may serve to gain insight into signals that govern β-cell differentiation and aid in developing culture systems to commit other (pluripotent) stem cells to clinically useful β-cells for cell therapy of T1D.

## Introduction

Type 1 diabetes (T1D) is caused by the selective loss of pancreatic β-cells by autoantigen-reactive T cells. The only way to permanently restore normoglycemia in T1D is by β-cell replacement through transplantation of an intact pancreas or isolated islet cells [1]. However, shortage of donors is one of the major limiting factors for treatment of T1D. Therefore, many groups are evaluating whether β-cells differentiated from stem cells could be an alternative cell source for β-cell replacement in T1D patients.

The pancreas is derived from definitive endoderm (DE), that specifies from pluripotent cells in the blastocyst stage of the embryo by a two-step process, wherein mesendoderm (ME) is generated to the exclusion of ectoderm, followed by specification to CXC chemokine receptor type 4 (Cxcr4) and SRY-related HMG-box (Sox)17 expressing DE [2]. Specification to pancreatic endoderm is associated with expression of Pancreatic and duodenal homeobox 1 (Pdx1). The expression of Pdx1 is regulated by the upstream transcription factor (TF) Hepatocyte nuclear factor (Hnf)6 [3], that also stimulates expression of the pro-endocrine gene, Neurogenin (Ngn)3 [4]. Other TFs important for β-cell differentiation include Paired box gene (Pax)4, that specifies endocrine pancreatic cells to a β-cell [5], NK6 homeobox (Nkx6).1 that regulates β-cell development [6]. Musculo aponeurotic fibrosarcoma oncogene homolog A (MafA) is expressed initially at e13.5 and is found only in insulin-positive cells during development or in mature islets. MafA is thought to act in conjunction with other known insulin enhancer regulatory factors (Neurogenic differentiation 1 (NeuroD1) and Pdx1) to promote transcription of the insulin gene [7].

Pancreas versus liver specification in the foregut is at least in part determined by Bone morphogenetic protein (BMP)4 and Fibroblast growth factors (FGF)2 produced by the adjacent cardiac mesoderm [8], [9]. Pancreas commitment from ventral as well as dorsal foregut endoderm is inhibited by Sonic hedgehog (SHH). Activin-A and FGF2 represses SHH expression in pre-pancreatic endoderm and facilitates endoderm formation [10]. Factors that guide final differentiation to β-cells has also been identified, being the most important Epidermal growth factor receptor (Erb)B1-3 [11], as well as Epidermal growth factor (EGF), Transforming growth factor (TGF)β, heparin-binding EGF, betacellulin (BTC) [12], and Growth and differentiation factor (GDF)11 [13], [14]. Exendin-4, a long-acting analogue of glucagon like peptide-1, up-regulates the expression of Pdx1 in human fetal islet clusters [15].

A number of studies have tested if embryonic stem cells (ESC) can be guided *in vitro* to β-cell like cells that would then be suitable for treatment of DM [16]–[20]. These studies have shown that although definitive endoderm and pancreatic endoderm commitment is readily achievable, full maturation towards functional, single insulin-positive β-cells *in vitro* remains difficult [21]. Nevertheless, some studies have shown that grafting of the partially committed and mixed m/hESC progeny in hyperglycemic mice can reverse diabetes after several weeks, even though in a number of studies teratoma formation was found [19], and in other studies, chiefly exocrine pancreatic tissue was found rather than endocrine pancreatic cells [21].

We described that multipotent adult progenitor cells (MAPC) isolated from rat bone marrow (rBM), can -like m/hESC- be guided to the hepatocyte-lineage, by sequential specification to ME, DE, hepatic endoderm and then hepatocytes [22], [23]. This formed the basis for studies described here wherein we tested if these cells can also be specified to insulin-secreting β-cells. Aside from evaluating rMAPC, we also evaluated the differentiation potential of rat extra-embryonic progenitor cells (rXEN-P) [24], isolated directly from rat blastocysts using rMAPC culture conditions (termed hypoblast stem cells or rHypoSC) [25]. Like rMAPC [25], [26], rHypoSC are a homogenous population of SSEA1/CD31-positive cells that express aside from Oct4 also a number of nascent hypoblast genes including FoxA2, Gata binding protein (Gata)6, Gata4, and Hnf1β, but not Nanog or Sox2, typical for ESC, caudal type homeobox 2 (Cdx2), typical for trophoblast or Hnf4α and αFetoprotein (αFP), typical for primitive endoderm (PrE). Like rMAPC, rHypoSC differentiate robustly to mesodermal cells and hepatic endodermal cells.

We here describe that two different cell lines of both rMAPCs and rHypoSCs can be committed to β-cell like cells using a four step protocol. This yields mixed progeny that contains a fraction of glucose responsive β-cell like cells and that reverses hyperglycemia upon grafting under the kidney capsule of nude streptozotocin (STZ)-treated mice.

## Materials and Methods

### Mice

Nude Balb/C mice were bred and housed in pathogen-free animal facilities at the University of Navarra. All the mice had free access to water and food. Animal procedures were performed following the criteria outlined in the Guide of the Care and Use of Laboratory Animals by the National Academy of Sciences. All procedures were approved by the animal experimentation ethics committee of the University of Minnesota, the Katholieke Universiteit Leuven and the University of Navarra (Permit Number: CEEA/105-10). All surgical procedures were performed under isoflurane anesthesia, and all efforts were made in order to minimize suffering.

### Cell isolation and culture

MAPCs were isolated from BM of Fisher rats (strain F344/IcoCrl) (Charles River, Wilmington, MA, USA). 1 to 4 week-old rats were used for isolation as described in detail in a protocol paper that was published recently [27]. Two rMAPC lines rMAPC-1 and Clone 19 (CL-19) were used in this study. For the isolation of blastocyst-derived XEN-P cells under MAPC culture conditions, in brief, blastocysts obtained from the Fisher rats were plated on flat-bottom Nunc 4-well plates (1 blastocyst/well, 0.5 ml medium/well) under rMAPC culture conditions. After 2–10 cells, cells with refractile morphology appeared, which could be expanded into cell lines by passaging on fibronectin-coated 100-mm dishes in rMAPC expansion medium. The rHyPoSc lines Fi2 and Wk8 were used in this study [25].

### Cytokines and growth factors used

The following cytokines and growth factors (all from R&D Systems unless mentioned) were added for cell expansion or during differentiation: rh/m/r Activin-A (338-AC), rhBMP4 (314-BP), mLIF (Millipore, ESG1107), rhFGF2 (233-FB), Cyclopamine (Biomol Research Lab, GR-334), betacellulin (1025-CE), Nicotinamide (Sigma, N0636), Exendin-4 (Sigma, E7144), GDF-11 (1958-GD), rhHGF (294-HGN), hPDGF-BB (220-BB), mEGF (R&D Systems 2028-3G) and monoclonal anti-human/mouse SHH N-terminal peptide antibody (MAB 4641).

### In vitro pancreas differentiation protocol

**Figure 1:**
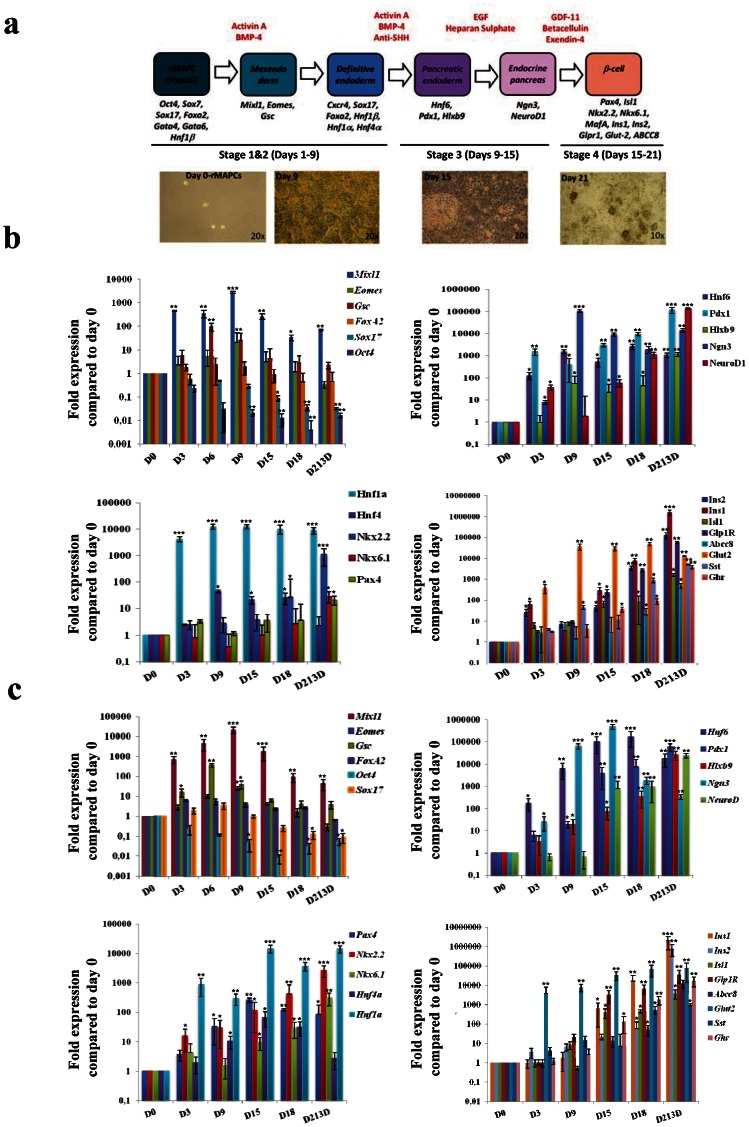
Gene expression profile of rMAPC and rHypoSC progeny at different stages during the differentiation protocol. (**A**) Schematic representation of the four stage pancreas differentiation protocol and the genes expected to be expressed at each stage of the protocol. In addition, light microscopy pictures of cell morphology at the end of each step of differentiation. RT-qPCR results of rMAPC-1 (**B**) and rHypoSC line Fi2 (**C**) progeny for ME/DE marker genes (*Mixl1*, *Eomes*, *Gsc*, *FoxA2*, *Sox17*, and *Oct4*) endoderm gene markers (*Hnf1β*, *Hnf4α*, *Nkx2.2*, *Nkx6.1* and *Pax4*) pancreas progenitor gene markers (*Hnf6*, *Pdx1*, *Hxbl9*, *Ngn3*, *NeuroD1*) and Islet genes (*Isl1*, *Ins1*, *Ins2*, *Glp-1R*, *ABCC8*, *Glut2*, *Sst*, *Ghr*) at different time-points during the pancreas differentiation protocol. Results are represented as mean ± SE from n = 3 independent differentiations. * p<0.05; ** p<0.01; ***p<0.001.

The pancreas differentiation protocol was carried out in four steps as shown in Figure 1A. Differentiation medium contains 60% DMEM low glucose, 40% MCDB, 2% fetal bovine serum, 1×ITS+1 (Sigma, I2521), 0.1 µM ascorbic acid 2-phosphate, 50 µM 2-mercaptoethanol, 100 units of penicillin, and 1000 units of streptomycin (all from Gibco BRL).

#### Step 1 [day 1 to 3]

rMAPCs or rHypoSCs were plated at 55,000–65,000 cells/cm^2^ on 24-well plates coated with 2% matrigel (BD) in expansion medium. 6–12 h later, medium was removed, cells were washed twice with PBS (Lonza), and differentiation medium containing activin A (100 ng/mL) and BMP-4 (50 ng/mL) was added.

#### Step 2 [day 3 to 9]

On day 3, media was exchanged for differentiation medium containing activin A (100 ng/mL), BMP-4 (50 ng/mL), and anti-SHH (2.5 mg/mL).

#### Step 3 [day 9 to 15]

On day 9, cells were washed twice with PBS and differentiation medium containing EGF (50 ng/mL) and heparan sulfate (50 ng/mL) (Sigma, H9902) was added.

#### Step 4 [day 15 to 21]

On day 15, cells were washed twice with PBS and differentiation medium with Exendin-4 (10 nM), betacellulin (50 ng/mL) and GDF-11 (50 ng/mL) was added. On day 18, a half media change was performed. To preserve the floating clusters in the culture, discarded medium was centrifuged (300 g, 5 min), cells were recovered and added again to the wells together with fresh medium.

### Real-time quantitative PCR (RT-qPCR)

RNA was obtained using the RNeasy Mini extraction kit (Qiagen, 74104) according to the manufacturer's instructions. The RNA extracted was quantified and 1 µg RNA/reaction was reverse transcribed to cDNA synthesis using Superscript III First-Strand synthesis system (Invitrogen 18080-051) with the following reaction conditions: first step, 25°C for 10 min; second step, 48°C for 30 min; and third step, 95°C for 5 min. The cDNA obtained underwent 40 rounds of amplification (Eppendorfrealplex/ABI 7000 (Eppendorf)) with the following reaction conditions: 40 cycles of a two-step polymerase chain reaction (PCR; 95°C for 15 min, 60°C for 60 min) after initial denaturation (95°C for 10 min) with 2 µl of cDNA solution (20 ng), 1×SYBR Green Platinum SYBR green qPCRSupermix-UDG (Invitrogen 11733-046) and 200 nM of each primer in a 12 µl total volume reaction. mRNA levels were normalized using GAPDH as housekeeping gene (TaqMan Rodent GAPDH Control Reagent, Applied Biosystems). List of primers used in this study has been mentioned in Table S4.

### FACS analysis of Pdx1 protein expression

For flow cytometry analysis (FACSAria, BD Biosciences), cells were harvested and washed twice with PBS and fixed for 10 min at room temperature in 4% paraformaldehyde (PFA, Sigma) in PBS. After fixation, cells were washed twice again with PBS. Later, in order to permeabilize the nuclear membrane, the cell pellet was resuspended in a PBS buffer containing 0.1% of saponin (Sigma, S7900) plus 0.4% of Fish Skin Gelatin (FSG; Sigma, G7765). Primary antibodies were diluted in PBS+0.4% FSG and incubated for 20 minutes at room temperature. Secondary antibodies were diluted in PBS+0.4% FSG and incubated for 15 minutes at room temperature. The following antibodies and dilutions were used: rabbit anti-Pdx1, 1∶1,000 (a kind gift from Dr.Wright, Vanderbilt University), AlexaFluor 594 anti-rabbit, 1∶500 (Molecular Probes, A11072).

### Western blot analysis

β-cell like clusters derived from rMAPCs were harvested on day 21 of differentiation by centrifugation (300 g, 5 min) and washed with PBS twice. Cell pellet was resuspended in lysis buffer (10 mM TrisHCl pH 8.0, 150 mMNaCl, 1 mM EDTA, 1% NP40, 0.5% deoxycholate, 0.1% SDS) supplemented with a cocktail of protease inhibitors (Roche) and 100 mM of phenylmethanesulfonyl fluoride (PMSF, Roche). Proteins were separated on Bis-Tris polyacrylamide gels, transferred by electroblotting onto PDVF membranes (BioRad) and detected using an horseradish-peroxidase conjugated secondary antibodies and chemiluminescent (ECL Plus, Amersham) exposure of BioMax film (Kodak). For the primary antibody, a rabbit anti-rat Pdx1 antibody was used a dilution of 1∶5,000–1∶10,000.

### C-peptide release assays

Rat C-peptide levels in culture supernatants or cell lysates were measured using the specific and ultrasensitive C-peptide ELISA from Wako (Code No. 295–57401, Richmond, VA USA). C-peptide release was measured by incubating either rMAPCs clusters (day 21 after initial differentiation), rat islets or rat insulinoma cell line (RIN) in Krebs-Ringer bicarbonate buffer (KRBB) containing 120 mM NaCl, 5 mM KCl, 1.1 mM MgCl_2_, 25 mM NaHCO_3_, and 0.1% bovine serum albumin. First hour incubation with KRBB solution was used as a washing step; medium was discarded and then we performed one hour incubation in KRBB containing 5 mM D-glucose or different stimulation conditions: 20 mM D-glucose, 10 mM D-glucose, 5 mM D-Glucose plus carbachol (100 µM: Sigma, C4382) or 20 mM D-glucose plus nifedipine (50 µM; Sigma, N7634) or 10 mM L-Arginine (Sigma). After one hour incubation, KRBB samples were stored at −20C. For C-peptide intracellular content, C-peptide was extracted from the cells with acid ethanol (10% glacial acetic acid in absolute ethanol) overnight at −20C followed by cell sonication. Both C-peptide release and C-peptide intracellular content were assayed using C-Peptide ELISA kit (Wako, Japan) according to the manufacturer instructions.

### Immunocytochemistry

For immunostaining, cells were washed twice with PBS and fixed for 10 min at room temperature in 4% paraformaldehyde (PFA) (Sigma) in PBS. After fixation, cells were washed three times with PBS. Cells were permeabilized for 30 minutes using PBS containing 0.2% Triton X-100 (PBST) (Acros Organics, New Jersey) to permeabilize the nuclear membrane. To block unspecific staining, cells were incubated 30 min in PBS containing 0.4% of Fish Skin Gelatin (FSG; Sigma). Primary antibodies were diluted in Dako antibody diluent (Dako, Glostrup, Denmark) and incubated overnight and were further washed with PBST and were incubated with secondary antibody for 1 hour at room temperature. 1 µg/ml Hoechst 33258 (Sigma-Aldrich) was used for nuclear staining.

For graft analysis, paraffin sections (5 µm thick) were rehydrated using standard procedures and fixed using 10% Neutral Buffered Formalin (NBF) for 15 minutes at room temperature and was permeabilized using PBST (Acros Organics). Nonspecific blocking was carried out with 10% serum corresponding to the animal source of secondary antibody (Dako) for 30 minutes. Primary antibodies were diluted with Dako antibody diluent and the cells were incubated overnight at 4°C followed by incubation with secondary antibodies conjugated with Alexa dyes (Invitrogen) for 1 hr at room temperature. Hoechst 33258 (Sigma-Aldrich) was added along with secondary antibody incubation for nuclear staining.

Following are the secondary antibodies and dilutions used in this study: goat anti-rabbit AlexaFluor 594, 1∶500 (Invitrogen, Carlsbad, CA); rabbit anti-goat AlexaFluor 594, 1∶500 (Invitrogen); goat anti-mouse AlexaFluor 594, 1∶500 (Invitrogen); goat anti-rabbit AlexaFluor 488, 1∶500 (Invitrogen); rabbit anti-goat AlexaFluor 488, 1∶500 (Invitrogen); goat anti-mouse AlexaFluor 488, 1∶500 (Invitrogen).

### Intraperitoneal glucose tolerance test (GTT)

Transplanted (rMAPC, n = 6, rHypoSc, n = 4) and non-transplanted (n = 4) nude Balb/C male mice were fasted overnight (16–18 h), weighed, and injected with D-glucose (Sigma) at a dose of 2 g/kg body weight intraperitoneally. Blood samples (50 µL) were obtained from the tail vein before and at 5, 10, 15 20, 25, 30, 45, 60, 75, 90 and 120 minutes after glucose administration. Blood glucose levels were analyzed by using a Glucometer Elite XL (Bayer).

### Cell transplantation

Experimental diabetes was induced in 8- to 10-week-old male nude Balb/C mice by a single intravenous injection of STZ (220 mg/kg of body weight) (Sigma) freshly dissolved in 0.1 M of citrate buffer, pH 4.5 (Sigma). Stable hyperglycemia (blood glucose levels 450–600 mg/dL) usually developed 48 to 72 hours after STZ injections. In addition, insulin-pellets (Linco, Canada) were implanted subcutaneously to prevent excessive glucose levels.

rMAPC or rHypoSC derived β-cell like cell clusters were collected on day 21 of differentiation by centrifugation (300 g, 5 min) and washed with PBS twice. 5×10^5^ differentiated cells were implanted under the left kidney capsule of ketamine/xylazine-anesthetized STZ-treated mice. The weight of animals was followed daily as well as the glucose concentrations, measured on blood collected from the tail vein using a Glucometer Elite XL (Bayer). Once animals had become normoglycemic, grafts were removed by unilateral nephrectomy. Before and after nephrectomy, blood samples were obtained to determine the C-peptide levels (Wako, Japan).

### Statistics

At appropriate places results are expressed as mean fold change ± S.E.M. The Gaussian distribution was confirmed using the Shapiro-Wilk test. In experiments where data differences between two groups were assessed, statistical significance was analyzed using the Student's unpaired t test. In order to compare the means of multiple groups ANOVA test was performed. For non-parametric data, the Kruskal-Wallis and Mann-Whitney tests were used. All statistical analyses were performed using the GraphPad Instat software version 3.00 for Windows (GraphPad Software, San Diego, CA, USA). All p values <0.05 were considered statistically significant.

## Results

### Signals required for induction of *Pdx1* and *Ins gene* expression in rMAPC-1

To develop a protocol for rMAPC/rHypoSC differentiation to β-cells, we initially used the rMAPC-1 cell line. We first tested the effect of cell density, different types of extracellular matrix coating and different concentrations of growth factors or inhibitors known to affect endoderm specification, including bFGF, activin-A, BMP4, retinoic acid (RA), cyclopamine and anti-SHH antibodies (Ab) alone or in combinations, on the induction of *Pdx1* expression on d6 in the rMAPC-1 cell line [22], [26]. *Pdx1* mRNA not present in undifferentiated rMAPC, was optimally induced on d6 when rMAPC were plated on matrigel and exposed for 6 days to 100 ng/mL activin-A and 50 ng/mL BMP-4, combined with 10 mM cyclopamine or 2.5 mg/mL anti-SHH Ab from d3-d6 (Figure S1A).

Subsequently, d6 committed rMAPC progeny were cultured for an additional 6 days with either EGF, heparan sulphate, FGF7, FGF10 or BTC. Only when cultured in the presence of EGF and heparan sulfate did we detect a further 4-fold increase in *Pdx1* transcript levels by d12 (Figure S1B).

To induce final maturation to insulin-expressing cells, we subsequently exposed on d12 rMAPC progeny to nicotinamide, exendin-4, GDF11 and/or BTC-all considered to be pancreatic β-cell maturation factors, alone or in combination. We evaluated the effect by measuring transcripts for *Pdx1* as well as *Insulin* (*Ins*)*1* and *Ins2* on d18. Transcript levels of *Pdx1* and *Ins2* were highest when GDF-11, BTC and Exendin-4 were added. However, *Ins1* levels were similarly induced when the following combinations were used: GDF11 and nicotinamide; GDF11, nicotinamide and betacellulin or GDF11, exendin-4 and betacellulin (Figure S1C) for the last 6 days of differentiation.

Further optimization was obtained by adjusting the duration of the different steps along the differentiation. As we observed extensive cell death when cyclopamine was added between d3 and d6, but not with anti-SHH Ab. Subsequent studies employed anti-SHH Ab that supported the differentiation process similarly to cyclopamine.

### rMAPC lines differentiate towards endocrine islet cells by a sequence of developmentally predicted steps

All subsequent studies were done using two previously described lines, CL19 and rMAPC-1 [22], [26], [27]. The protocol designed in the present study is represented schematically in Figure 1A and in detail in material and methods. Under this standardized conditions, small areas of epithelioid cells could be detected from d9. By d19 (step 4), these epithelioid patches detached to form clusters of 50–150 cells floating in the culture supernatant. Therefore, on d21, RT-qPCR analysis was performed on adherent cells and on the clusters in the suspension.

We followed the transcript levels for genes expressed in ME/DE, pancreatic endoderm, endocrine pancreatic endoderm and mature islets by RT-qPCR on d3, d6, d9, d15, d18 and d21 (for rMAPC-1 see Figure 1B and Table S1, for CL19, see Table S2). Transcripts for the ME/DE marker genes, *Goosecoid* (*Gsc*), *Eomesodermin* (*Eomes*) *Mix1 homeobox-like 1*(*Mixl1*) and *Cxcr4* were induced maximally between d6 and d9, and decreased upon removal of Activin-A. *Foxa2* and *Sox17*, already present in undifferentiated rMAPC, persisted throughout the culture. Expression of other endoderm TFs, *Hnf1α* and *Hnf4β*, increased progressively during differentiation, whereas levels of *Hnf1β*, already high in rMAPC, persisted throughout differentiation (Figure 1B and Tables S1 & S2).

Transcript levels of *Hnf6* increased from d3 of differentiation, persisting till d21 (Figure 1B). Consistent with the increase in *Hnf6*, *Pdx1* expression became detectable from d3 onwards reaching levels in the d21 non-adherent clusters approaching 10% of those found in mature β-cells. *Hlxb9* transcript levels progressively increased till d21, to levels 10 fold higher than in mature β-cells. *Ngn3*, was induced from d9 onwards, and persisted also in the d21 non-adherent clusters. Expression of *NeuroD1* increased from d18 onwards, reaching maximal levels on d21 in the non-adherent clusters. *Pax4* and *Nkx2.2* transcripts rose from d15 onwards and *Nkx6.1* from d18 in the 3D clusters to levels similar to those in β-cells (Figure 1B and Tables S1 & S2).

*Ins1* and *Ins2* mRNA became detectable on d15, reaching levels of 0.1% of β-cells in the non-attached clusters on d21 (Figure 1B). We could not detect *glucagon* (*Gcg*) transcripts, but transcripts for *Somatostatin* (*Sst*) and *Ghrelin* (*Ghr*) increased on d18 and d21, respectively, to levels 0.08% and 0.1% of mature islets. Day 21 progeny also expressed *insulin gene enhancer protein* (*Isl1*) and *ATP-binding cassette transporter sub-family C member 8* (*ABCC8*) transcripts at levels near those in β-cells, whereas levels of *Glucose transporter 2* (*Glut2*) and *Glucagon-like peptide 1 receptor* (*GLP1R*) were between 1 and 5% this in primary β-cells. Transcripts for *amylase* were not detected (Figure 1B and Tables S1 & S2).

It should be noted that aside from pancreatic endoderm and more mature β-cell transcripts, transcripts coding for *alpha-fetoprotein* (*αFP*) were induced from d3 onwards, transcripts for the PrE TF *Sox7* persisted throughout differentiation, while transcript levels of *albumin* increased significantly less. *αFP* levels were, however, significantly lower in d21 non-adherent clusters compared with earlier time points. *VE-Cadherin* and *Flk1* were expressed from d9 and d18 onwards and were also expressed in the non-adherent clusters (Tables S1 & S2 and Figure S3).

**Figure 2:**
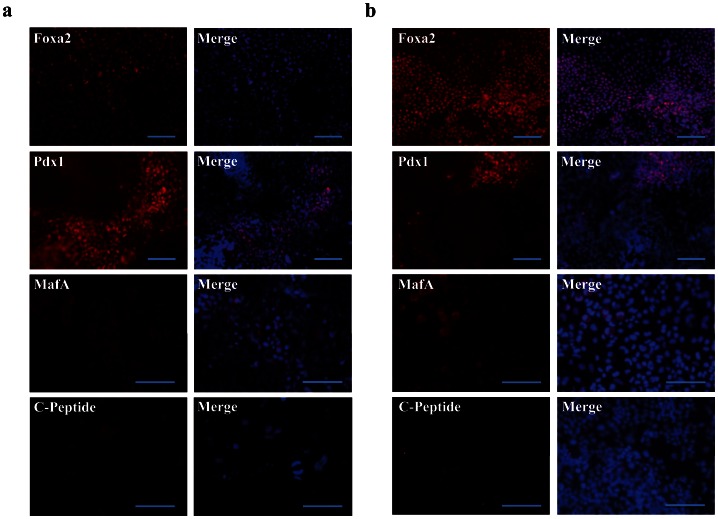
Immunostaining of rMAPC and rHypoSC progeny. Fluorescence micrographs showing expression of FoxA2, Pdx1, MafA and C-peptide in d21 rMAPC-1 (**A**) and Fi2 (**B**) differentiated progeny. Scale bars, 100 µm. Representative for n = 3 independent differentiations. Merge indicate FoxA2, Pdx1, MafA and C-peptide together with DAPI staining.

Immunostaining demonstrated that on d21, 84%±4 expressed FoxA2, 21%±3 Pdx1, 14%±1 MafA and 7%±1 C-peptide, although the staining intensity of C-peptide per cell was low (Figure 2A). Of note MafA expression appeared to be more perinuclear than nuclear, consistent with the notion that the cells generated were immature β-cell like cells [28]. Presence of Pdx1-positive cells in the non-adherent clusters on d21 was confirmed by FACS (Figure S2A; average percent positive cells 25%±0.07), and western blot (Figure S2B).

### Like rMAPCs, rHypoSC also differentiate into insulin-expressing cells

As rHypoSC have a similar gene expression profile and differentiation ability *in vitro* compared with rMAPC [25], we tested if the protocol used for rMAPC would also induce commitment of blastocyst-derived rHypoSC towards pancreatic endoderm and β-cell like cells *in vitro*. Studies were done using two independently derived cell lines, Fi2 and WK8. Like rMAPC, commitment of rHypoSC occurred via the initial activation of ME/DE genes, followed by sequential activation of pancreatic and endocrine pancreatic transcripts, among others *Hnf6* from d3 onwards, *Pdx1* from d9, and *Ngn3*, *NeuroD1* and *Nkx6*.*1* from d15 onwards (see Figure 1C and Table S3). The expression pattern of *Abcc8*, *Glut2*, and *Ins1* was similar to the expression pattern seen in rMAPC progeny (Figure 1C). Expression of FoxA2, Pdx1, MafA and C-peptide was confirmed at the protein level by immunostaining (Figure 2B).

### Differentiated rMAPC and rHypoSC progeny show regulated insulin secretion *in vitro*

**Figure 3:**
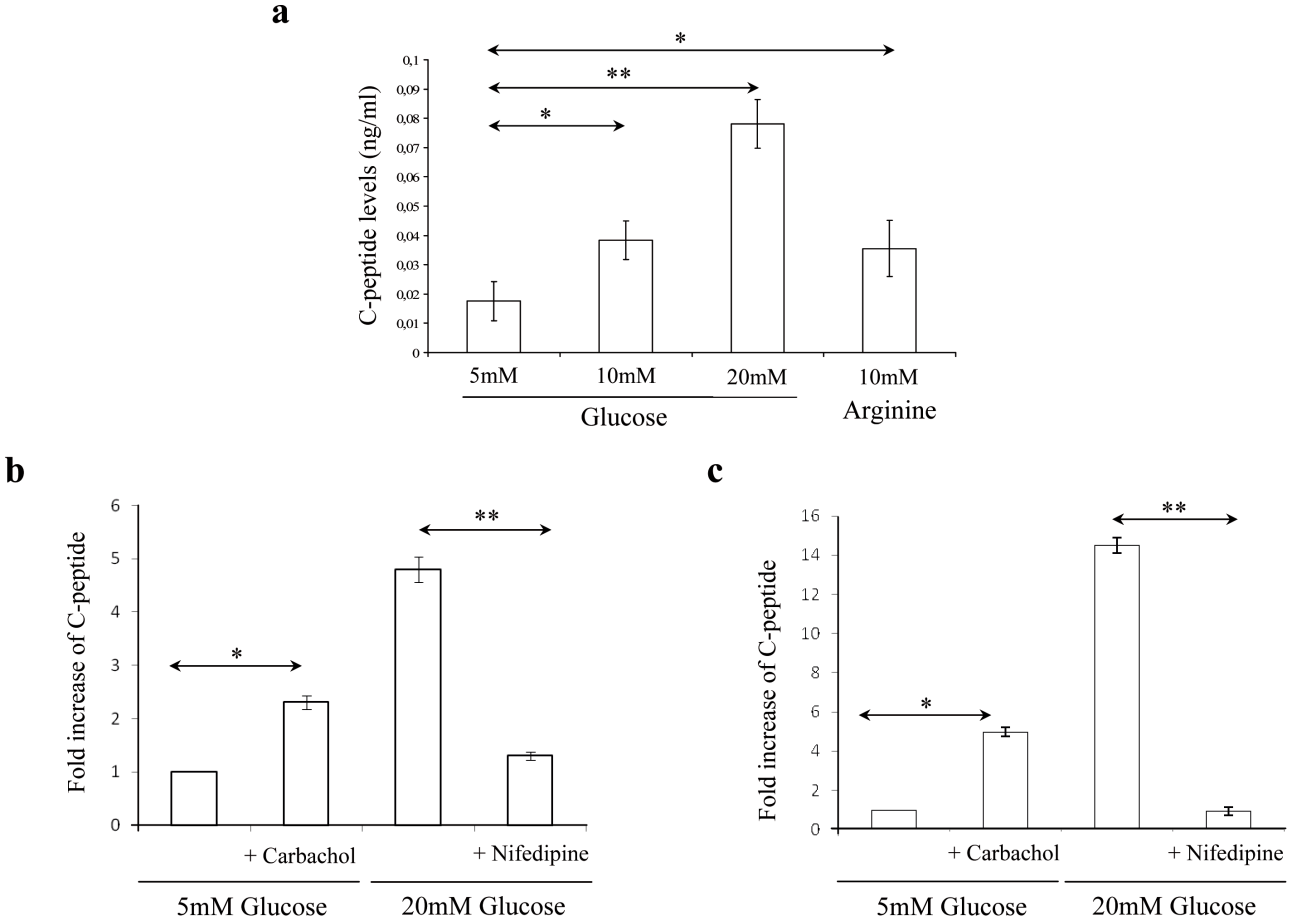
d21 rMAPC and rHypoSC derived progeny release C-peptide in response to glucose in a physiological manner. (**A**) C-peptide release in response to different glucose concentrations (5, 10 & 20 mM) and L-Arginine (10 mM) was measured after static incubation of differentiated rMAPC-1 for one hour. C-peptide release in response to carbachol (100 µM) in the presence of low glucose levels (5 mM) and in response to a inhibitor of insulin secretion, Nifedipine (50 µM), in the presence of high glucose (20 mM) stimulation were analysed in the differentiated rMAPC (**B**) and Fi2 (**C**). Results shown are mean ± SEM of 3 independent experiments including 3 replicates per experiment. * p<0.05; ** p<0.01.

We next determined if differentiated rMAPC and rHypoSC progeny secrete C-peptide in response to glucose. In an initial set of studies we stimulated rMAPC-1 progeny between d16 and d24 with a daily pulse of 20 mM glucose for 1 h and examined the kinetics of C-peptide release. The secreted C-peptide levels increased gradually and reached a peak level of 1.75 ng/mL on d21–22, coincident with the peak levels for *Ins1* and *Ins2* mRNA expression (Figure S4). C-peptide secretion increased significantly when differentiated rMAPC-1 were subjected to increased glucose concentrations (from 5 to 20 mM) on d21 or following stimulation with 10 mM of the aminoacid, L-arginine (Figure 3A).

We also examined the effect of the muscarinic cholinergic receptor agonist, carbachol [29], and L-type Ca^2+^ channel blocker, nifedipine [30], on insulin secretion. Carbachol stimulated C-peptide release in the presence of low concentration of glucose (5 mM), whereas nifedipine inhibited C-peptide secretion in the presence of high glucose concentrations (20 mM) in both rMAPC-1 and rHypoSC (Fi2) progeny (Figures 3B & 3C).

### rMAPC and rHypoSC progeny normalize blood glucose levels in streptozotocin (STZ)-diabetic mice

**Figure 4:**
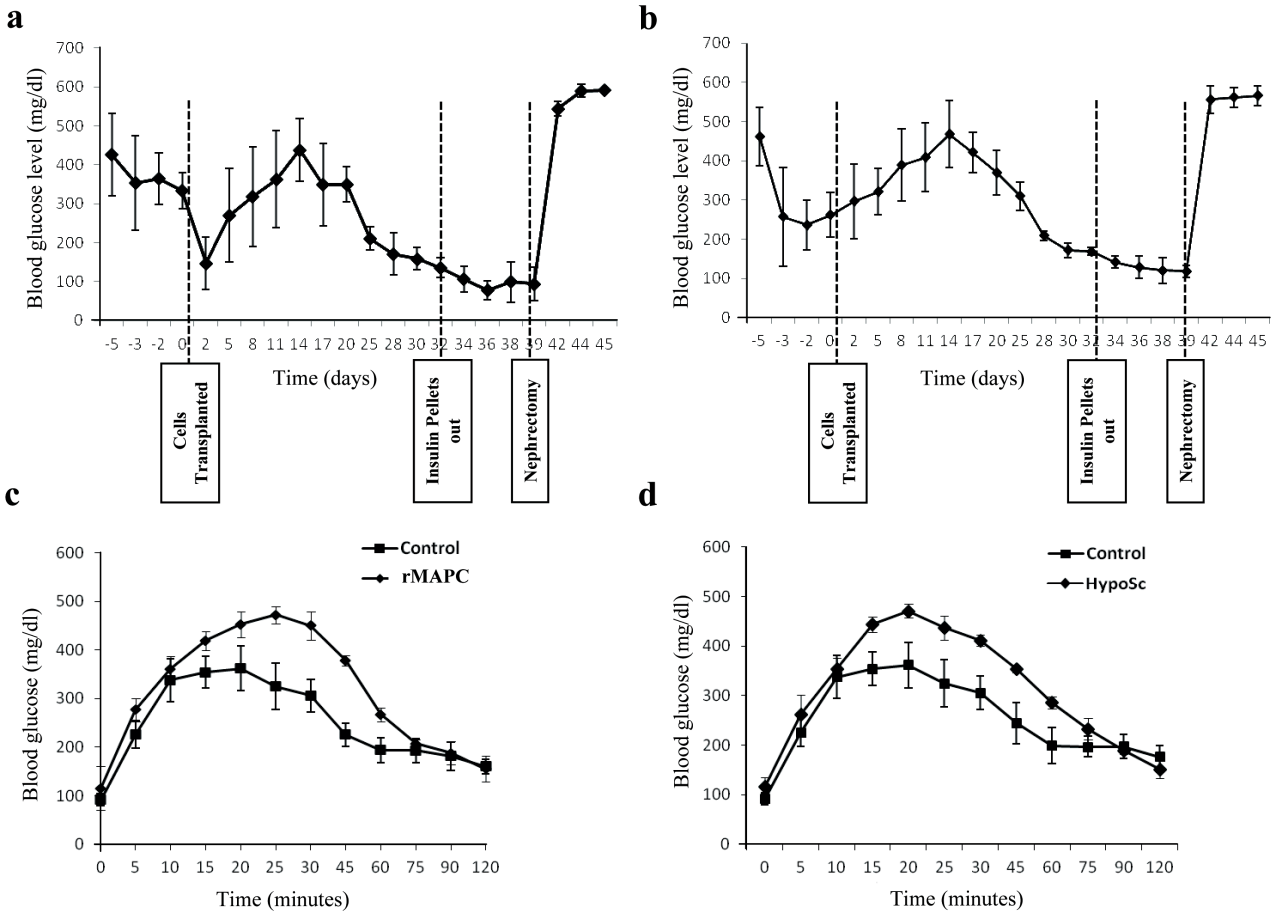
Reversal of hyperglycemia in the STZ treated mice. Reversal of sustained high blood glucose level in STZ-treated diabetic nude mice after renal subcapsular implantation of d21 non-attached clusters derived from rMAPC (**A**) and rHypoSC (**B**). 6 out of 10 animals transplanted with rMAPC differentiated to insulin-producing cells achieved normoglycemia (**A**) and 5 out 10 in rHypoSC group (**B**). Average of blood glucose levels in response to intraperitoneal glucose tolerance test for the mice transplanted with differentiated rMAPC (n = 6) (**C**) and rHypoSCs (n = 4) (**D**) and were compared with control mice (n = 4). Data shown are mean ± SEM of glycemias over time (panels A and B) and of glucose tolerance test (panel C and D).

We next tested if rMAPC and rHypoSC progeny could reverse hyperglycemia *in vivo*. Nude mice were injected with STZ to eliminate endogenous β-cells, and received an implantable insulin pellet to control excessive hyperglycemia. On day 7±2 following STZ injection, 5×10^5^ (rMAPC-1 n = 5; CL19 n = 5; Fi2 n = 5; WK8 n = 5) d21 non-adherent cells were grafted under the kidney capsule of the mice. The weight and blood glucose levels of mice were followed every other day after the cells were grafted (Figure S5). Once mice had become near normoglycemic (3 consecutive days of glycemia levels<120 mg/dL), insulin pellets were removed and animals followed for an additional 6 to 8 days. Subsequently, the engrafted kidney was removed and blood glycemia was further followed up (Figures 4A & 4B).

Six out of 10 and 5 out of 10 mice grafted with 5×10^5^ rMAPC and rHypoSC progeny, respectively, became normoglycemic between d25–30 and d30–35 respectively (Figures 4A and 4B). An intraperitoneal glucose tolerance test (GTT) was performed in mice that received rMAPC (rMAPC-1 n = 4; CL19 n = 2) and rHypoSC (Fi2 n = 2; WK8 n = 3) progeny once normoglycemia was achieved. A similar increase and clearance of glucose was seen as in control non-diabetic mice, in both recipients of rMAPC and HypoSC progeny (Figures 4C and D). Before nephrectomy, C-peptide levels in the serum were 23±1.9 ng/mL and 21±1.6 ng/mL in recipients of rMAPC or rHypoSC progeny, respectively. After unilateral nephrectomy, C-peptide levels decreased to that of STZ-treated mice without cell grafts (Figure S6), and all animals became hyperglycemic within one day (Figure 4A and 4B).

**Figure 5:**
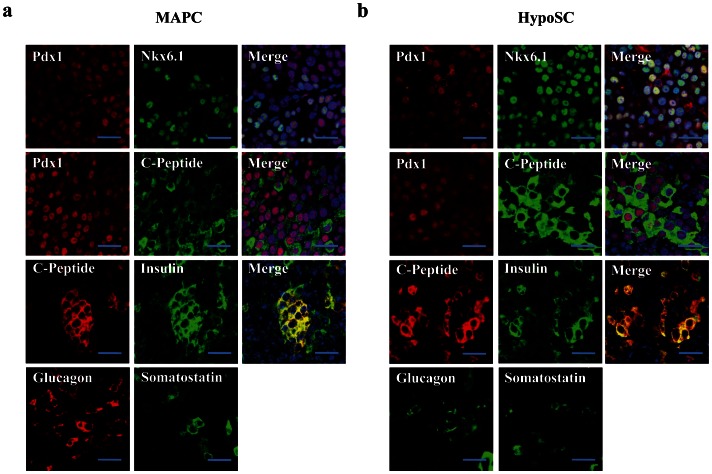
Immunofluorescence analysis of the graft. Micrographs from serial sections of the graft from the mice transplanted with differentiated rMAPC (**A**) and rHypoSC (**B**) were double stained for the analysis of colocalization for the pancreatic proteins Nkx6.1 and Pdx1, C-peptide and Pdx1, C-peptide and Insulin. All C-peptide positive were also insulin positive. Grafts were also analysed for the expression of other islet hormones, glucagon and somatostatin. Scale bar, 50 µm.

The grafts contained 61±9% and 70±11% Pdx1-positive cells, 56±6% and 64±7% of which co-expressed Nkx6.1 and 48±7% and 51±4% C-peptide, respectively in 5×10^5^ rMAPC or HypoSC progeny grafts (Figure 5). In addition, we detected 8±2% and 10±2% glucagon-positive cells and 2±0.8% and 3±0.7% somatostatin-positive cells in rMAPC or HypoSC progeny recipients (Figure 5). For 3±0.6% of glucagon and 1±0.8% of somatostatin cells, co-expression with C-peptide was seen in both rMAPC and rHypoSC (Figure S7). However, as there is no homogenous coexpression of PDX1 with NKX6.1 nor with C-peptide (Figure 5), we believe the graft is not yet completely matured as comparable to endogenous islets. Insulin and C-peptide were co-expressed indicating that the insulin we detect was *de novo* synthesized. Normal rat pancreas was used as a control for the analysis of the graft (Figure S8). In the graft we also analyzed for the presence of αFP and there were no αFP positive cells colocalizing with C-peptide were found *in vivo*.

## Discussion

A number of strategies are being explored to increase the β-cell mass as therapy of T1D, including among others regeneration of β-cells from intra-pancreatic (ductal) progenitor cells [31], transdifferentiation of α-cells to β-cells [32], or even transdifferentiation of hepatocytes to β-cells [33]. A number of studies have been published wherein mouse or human ESC and induced pluripotent stem cells (iPSC) can be guided *in vitro* towards DE, endocrine endoderm, or even near-mature β-cells that upon grafting in murine models of diabetes, can impact hyperglycemia [16]–[20].

Here we used rMAPC [22], [26], [27] and rHypoSC [25] cells isolated from rat blastocysts using MAPC culture conditions to generate endocrine pancreas and β-cell like cells. We have previously shown that rMAPC [22] and rHypoSC [25] can be fated towards hepatic endodermal cell types, apparently by refating the cells from a PrE to ME/DE fate. We demonstrate here that culture of rMAPC and rHypoSC with 100 ng/mL activin-A combined with 50 ng/mL BMP4 induces a complement of marker genes (*Gsc*, *Eomes*, *Mxl1*, *Cxcr4*, *Sox17*, and *FoxA2*) which, when combined, represents ME/DE. Compared with most published studies wherein activin-A is added for 3–4 days to commit ESC to DE, we found that stimulation for up to 9 days with activin-A was required to generate optimally DE. This could be consistent with the fact that rMAPC/HypoSC first have to switch from a more PrE-like phenotype to epiblast-like cells, thereby gaining the ability to undergo “gastrulation”. We have previously demonstrated a similar phenomenon when inducing rMAPC to hepatocyte-like cells [22]. It was commonly accepted that DE derivatives are solely derived from the epiblast. However, Kwon *et al.* demonstrated that aside from DE, a significant number of cells in the gut of 12–18 somite embryos are derived from visceral endoderm (VE), direct descendants from PrE [34]. *Sox7*, a TF typical for PrE and VE and highly expressed in rMAPC and HypoSC continued to be expressed during differentiation. In addition, although we expected *Glut2* transcripts to be expressed only once insulin-producing cells are being formed, we saw a significant increase in *Glut2* as early as 3 days after the differentiation protocol was applied. As this coincided with the induction of *αFP*, and *Glut2* has been described in VE [35], we believe that the very quick increase in *αFP* and *Glut2*, and persistent expression of *Sox7* may be due to default differentiation of a fraction of the rMAPC/HypoSC to VE. It should be noted that in contrast to *αFP* mRNA levels that were >100 fold lower in the non-attached clusters than the attached cells, *Glut2* transcripts remained expressed in the clusters. Hence, a fraction of the rMAPC/HypoSC default differentiated to VE, without this interfering with the generation of endocrine pancreas and eventually mature β-cell like cells. We detected also some induction of endothelial-cell specific genes throughout the differentiation, suggesting that rMAPC and HypoSC generated mesoendoderm, allowing mesodermal differentiation to follow.

The differentiation protocol used is in some aspects similar to the protocols used to specify mouse and human ESC or iPSC towards β-cells, even though significant differences are present as well. Similar to most differentiation protocols 16–20 initial commitment to the ME/DE required high concentrations of activin-A. Presence of fetal calf serum in our differentiation protocol is likely responsible for the fact that addition of *Wnt3a*, known to also play a role in ME/DE commitment [36], was not required. The first difference between our protocol and recently developed protocols for ESC/iPSC differentiation towards pancreatic endoderm is the positive effect noted with addition of BMP4 during the initial step of differentiation, whereas others have reported that inhibiting BMP4 signaling is required to specify DE to pancreatic and not hepatic endoderm [9], [18]. As discussed above, even though we found expression of *αFP* from d3 onwards, this is likely not a reflection of hepatic differentiation, but evidence of default differentiation towards visceral endoderm. It is possible that the difference in fate of rMAPC/HypoSC compared with ESC plays a role in the differential requirement for BMP4. Alternatively, species differences may be of importance, as Serafimidis *et al.* [37], also demonstrated the need for BMP4 in murine ESC specification towards pancreatic endoderm. It is well known that significant differences exist in the requirements for growth factors from the TGFβ family, and in particular BMP4, for ESC maintenance from different species. Hence, the requirement for BMP4 for lineage specification may also be species-dependent. The second difference between the protocol used for rMAPC/HypSC differentiation and published protocols are that we could not detect an effect of retinoic acid on pancreatic endoderm commitment.

As in most studies, the *in vitro* differentiated progeny from rMAPC and rHypoSC were mixed populations of “immature” β-cell like cells. We detected *Somatostatin*, *Ghrelin*, *Ins1* and *Ins2* transcripts, but could not detect *Glucagon* transcripts *in vitro*. Between 5 and 10% of the *in vitro* differentiated progeny expressed C-peptide. Such cells are likely still immature because MafA protein was expressed perinuclear, as has been described for neonatal rat β-cells [28]. Nevertheless, we demonstrated that the β-cell like cells in the d21 progeny secrete C-peptide in response to both glucose and L-arginine as well as in response to the addition of carbachol [29] and secretion is blocked by the Ca^2+^ antagonist nifedipine [30].

Final proof that functional β-cell like cells have been generated is demonstrated by studies wherein the grafted cells rescue animals that are hyperglycemic. Studies on islet from different species have recognized that the cytoarchitecture of pancreatic islets differs between species. In particular, rodent islets are composed of 80% β cells, 10% α cells, <8% δ cells, and <2% PP cells. Immunofluorescence analysis of rodent islets showed unique arrangement of hormone producing cells in that the insulin-expressing cells being clustered in the core of the islets and the non-beta-cells, including alpha-, delta- and PP-cells, form the mantle region (37). When 5×10^5^ islet cells (corresponding to approximately 500 mature islets) are grafted in STZ-diabetic mice, an immediate return to normoglycemia is observed. However, following grafting of 5×10^5^ rMAPC and HypoSC progeny, normoglycemia did not return for approximately 1 month. This is consistent with the fact that although the cells that were transplanted contained >90% FoxA2-positive-endoderm cells (±4.5×10^5^ cells) and 21% Pdx1-positive pancreatic endoderm cells (±10^5^ cells), they contained only 7% C-peptide-positive cells (0.35×10^5^ cells).

It is thus very likely that additional β-cell like cells needed were generated from the FoxA2 and/or Pdx1-positive fraction *in vivo* to eventually correct the hyperglycemia. Consistent with this, we could also detect glucagon-positive cells in the graft, but not in the *in vitro* generated progeny. Therefore we believe that FoxA2 and/or Pdx1-positive cells matured *in vivo*, generating cells expressing the three major endocrine hormones, even though a fraction of the cells still were C-peptide and glucagon double positive, and hence not yet fully mature (Figure S7). Nevertheless, the β-cell-like cells present in the graft *in vivo* could handle glucose in a physiological manner, as glucose disposal *in vivo* following glucose tolerance test was highly similar as in non-diabetic mice. Upon removal of the graft, wherein no tumors were found, all animals became hyperglycemic again.

In conclusion, we here demonstrate again that cells that have a nascent hypoblast phenotype, can *in vitro* be fated to generate intraembryonic endoderm (and mesoderm), via an intermediate ME/DE stage. This occurs combined with initial default differentiation towards visceral endoderm. Nevertheless, once committed to ME/DE, cells can be further specified to pancreatic endoderm and (immature) β-cell-like cells. When grafted *in vivo*, the mixed progeny transplanted under the kidney capsule of STZ-induced diabetic mice can reverse hyperglycemia, and this by producing physiological amounts of insulin for a given glucose load. Extension of this protocol to other “pluri”potent stem cells will be of interest. In addition studies aimed at isolating human HypoSCs may provide an alternative cell source that can relatively robustly differentiate into glucose responsive insulin-producing β-cell like cells.

## Supporting Information

Figure S1 **Standardization of the differentiation protocol.** **(A)** RT-qPCR analysis showing the expression of *Pdx1* after the stimulation of rMAPC-1 using different combination of cytokines. Only the combination of Activin-A (100 ng/mL) and BMP-4 (50 ng/mL), together with the inhibition of Shh, was able to up-regulate the expression of *Pdx1*. **(B)** Expansion of the *Pdx1* positive cells with EGF, HGF, FGF-10 or betacellulin (50 ng/mL) starting from rMAPC-1 cultured for 6-9 days with Activin A+BMP-4+anti-Shh (stages 1 & 2). **(C)** RT-qPCR results for *Pdx1* and *Ins1* & *Ins2* on rMAPC-1 cultured with different combinations of Nicotinamide (10 mM), Exendin-4 (10 nM), BTC (50 ng/mL) and GDF-11 (50 ng/mL) during the last step of differentiation. *Pdx1* and *Ins*1/2 mRNA levels are expressed in % versus a positive control (rat pancreas) and were normalized by using GAPDH as housekeeping gene. RT-qPCR results are expressed as mean (± SEM) of experiments performed in triplicates. * p<0.05; ** p<0.01; ***p<0.001.
(TIF)

Figure S2 **Pdx1 protein expression analysis on cell-clusters derived from rMAPCs.** **(A)** Intracellular FACS analysis showing the expression of Pdx1 in d 21 cell clusters generated by rMAPC-1. Pdx1 expression is compared to undifferentiated rMAPC and the insulinoma cell line RINm5F. **(B)** Confirmation of Pdx1 expression on d21 rMAPC cell clusters by western blot analysis.
(TIF)

Figure S3 **Expression analysis of hepatic and mesodermal lineage genes during the course of differentiation of rMAPC.** mRNA expression of hepatic **(A)** and mesodermal **(B)** lineage genes was quantified by RT-qPCR analysis. Data shown are the average of three independent experiments.
(TIF)

Figure S4 **C-peptide release by rMAPC progeny during the course of differentiation.** C-peptide secretion after one hour incubation with 20 mM glucose during the last 9 days (forth step+3 days) of the differentiation protocol was analyzed using rat C-peptide specific ELISA kit. The results shown are the average of three independent experiments. SEM±, n = 3 experiments. * p<0.05, ** p<0.01.
(TIF)

Figure S5 **Analysis of body weight of the grafted animals.** Body weight of mice transplanted with differentiated rMAPC (n = 6) and rHypoSC (n = 4) were monitored up to 45 days post transplantation. Data shown is mean ±SEM of the weight.
(TIF)

Figure S6 ***In vivo*** **C-peptide analysis in mice transplanted with differentiated cells.** Pre- and post-nephrectomized C-peptide release was measured in the serum of mice transplanted with differentiated **(A)** rMAPC (n = 6) and **(B)** rHypoSC (n = 4). Data shown is mean±SEM. ** p<0.01.
(TIF)

Figure S7 **Immunohistological analysis of the graft.** In some areas of the graft, colocalization of C-peptide and glucagon in both rMAPC and rHypoSC progeny was detected, indicating persistence of some immature hormone expressing cells. Scale bar = 50 µM.
(TIF)

Figure S8 **Immunohistological analysis of normal rat pancreas.** As a positive control for the antibodies used for staining, normal rat pancreas were stained for islet hormones C-peptide and insulin and for transcription factors PDX1 and NKX6.1 along with nuclear specific staining using DAPI. Scale bar = 50 µM.
(TIF)

Table S1 **Quantitative PCR analysis of genes expressed during the course of differentiation of rMAPC1 to β-cell like cells (n = 3 experiments, ±SD).**
(DOCX)

Table S2 **Quantitative PCR analysis of genes expressed during the course of differentiation of CL19 to β-cell like cells (n = 3 experiments, ±SD).**
(DOCX)

Table S3 **Quantitative PCR analysis of genes expressed during the course of differentiation of HypoSC to β-cell like cells (n = 3 experiments (Fi-1 = 2n and WK8 = 1n) ±SD).**
(DOCX)

Table S4 **Primer sequences used for the transcript detection by RT-qPCR.**
(DOCX)
