# Supplementary figures and images for: Reversal of Hyperglycemia by Insulin-Secreting Rat Bone Marrow- and Blastocyst-Derived Hypoblast Stem Cell-Like Cells
Source: PLoS One. 2013 May 9;8(5):e63491. doi: 10.1371/journal.pone.0063491 (PMC3650069; doi:10.1371/journal.pone.0063491)

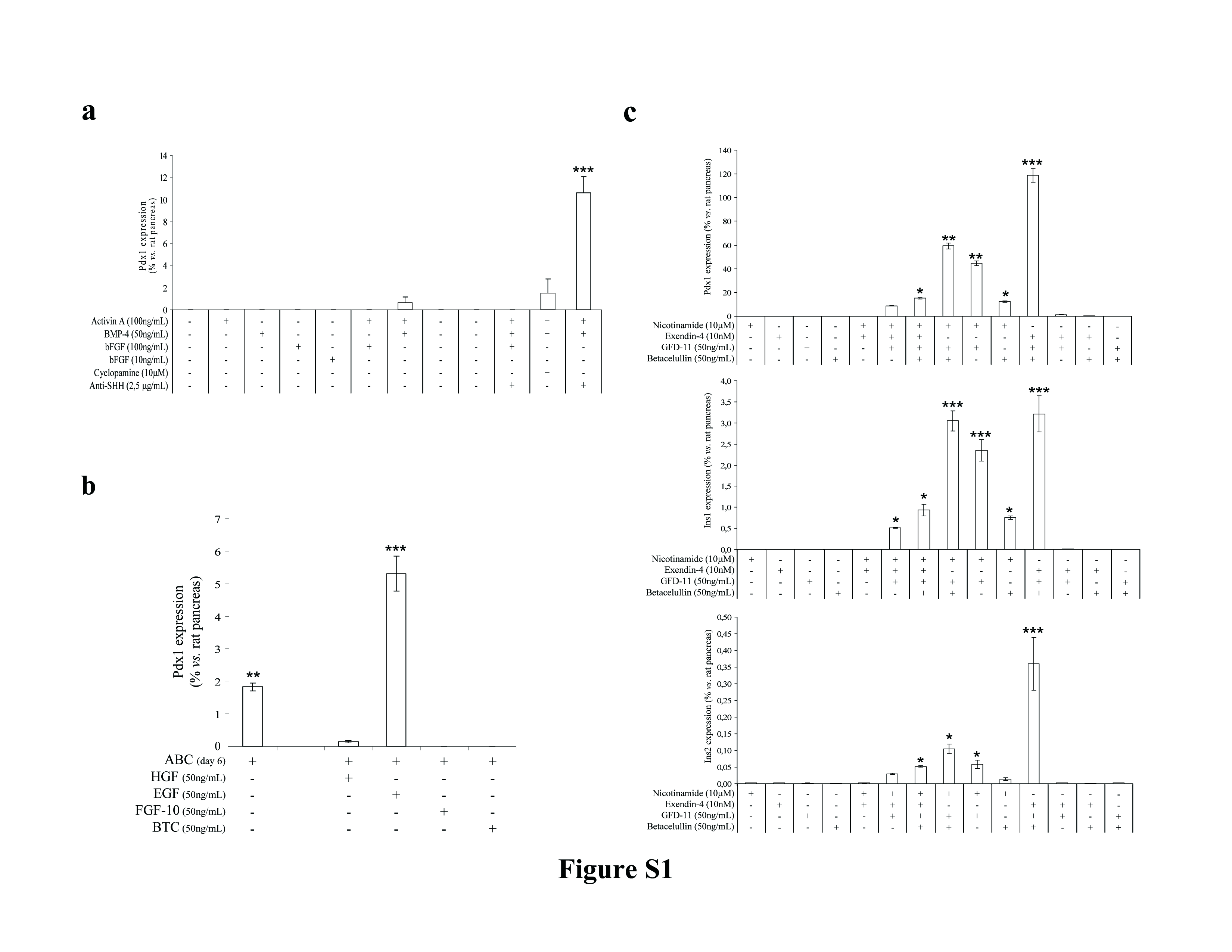

Supplement: Figure S1 — Standardization of the differentiation protocol. (A) RT-qPCR analysis showing the expression of Pdx1 after the stimulation of rMAPC-1 using different combination of cytokines. Only the combination of Activin-A (100 ng/mL) and BMP-4 (50 ng/mL), together with the inhibition of Shh, was able to up-regulate the expression of Pdx1. (B) Expansion of the Pdx1 positive cells with EGF, HGF, FGF-10 or betacellulin (50 ng/mL) starting from rMAPC-1 cultured for 6-9 days with Activin A+BMP-4+anti-Shh (stages 1 & 2). (C) RT-qPCR results for Pdx1 and Ins1 & Ins2 on rMAPC-1 cultured with different combinations of Nicotinamide (10 mM), Exendin-4 (10 nM), BTC (50 ng/mL) and GDF-11 (50 ng/mL) during the last step of differentiation. Pdx1 and Ins1/2 mRNA levels are expressed in % versus a positive control (rat pancreas) and were normalized by using GAPDH as housekeeping gene. RT-qPCR results are expressed as mean (± SEM) of experiments performed in triplicates. * p<0.05; ** p<0.01; ***p<0.001. (TIF) [file pone.0063491.s001.tif]

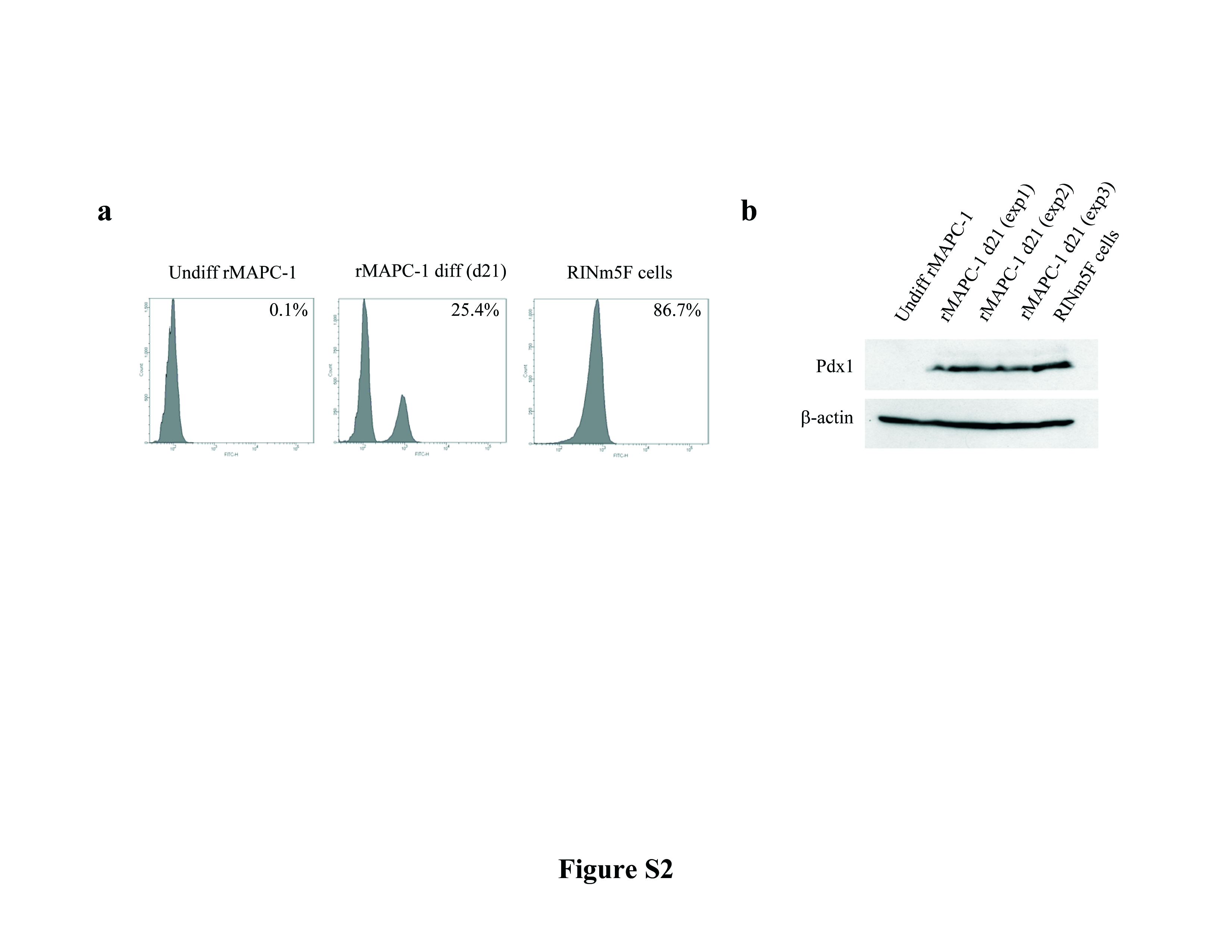

Supplement: Figure S2 — Pdx1 protein expression analysis on cell-clusters derived from rMAPCs. (A) Intracellular FACS analysis showing the expression of Pdx1 in d 21 cell clusters generated by rMAPC-1. Pdx1 expression is compared to undifferentiated rMAPC and the insulinoma cell line RINm5F. (B) Confirmation of Pdx1 expression on d21 rMAPC cell clusters by western blot analysis. (TIF) [file pone.0063491.s002.tif]

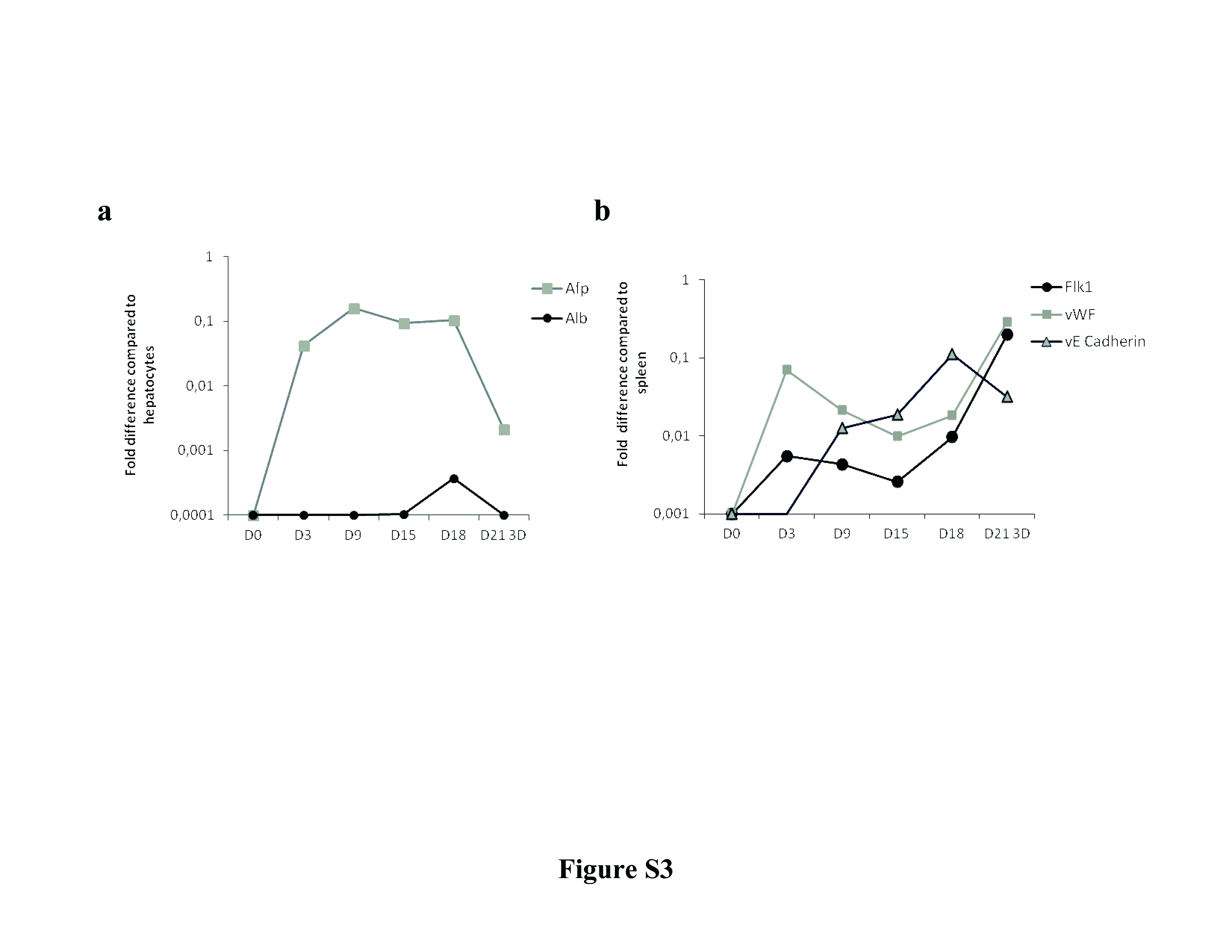

Supplement: Figure S3 — Expression analysis of hepatic and mesodermal lineage genes during the course of differentiation of rMAPC. mRNA expression of hepatic (A) and mesodermal (B) lineage genes was quantified by RT-qPCR analysis. Data shown are the average of three independent experiments. (TIF) [file pone.0063491.s003.tif]

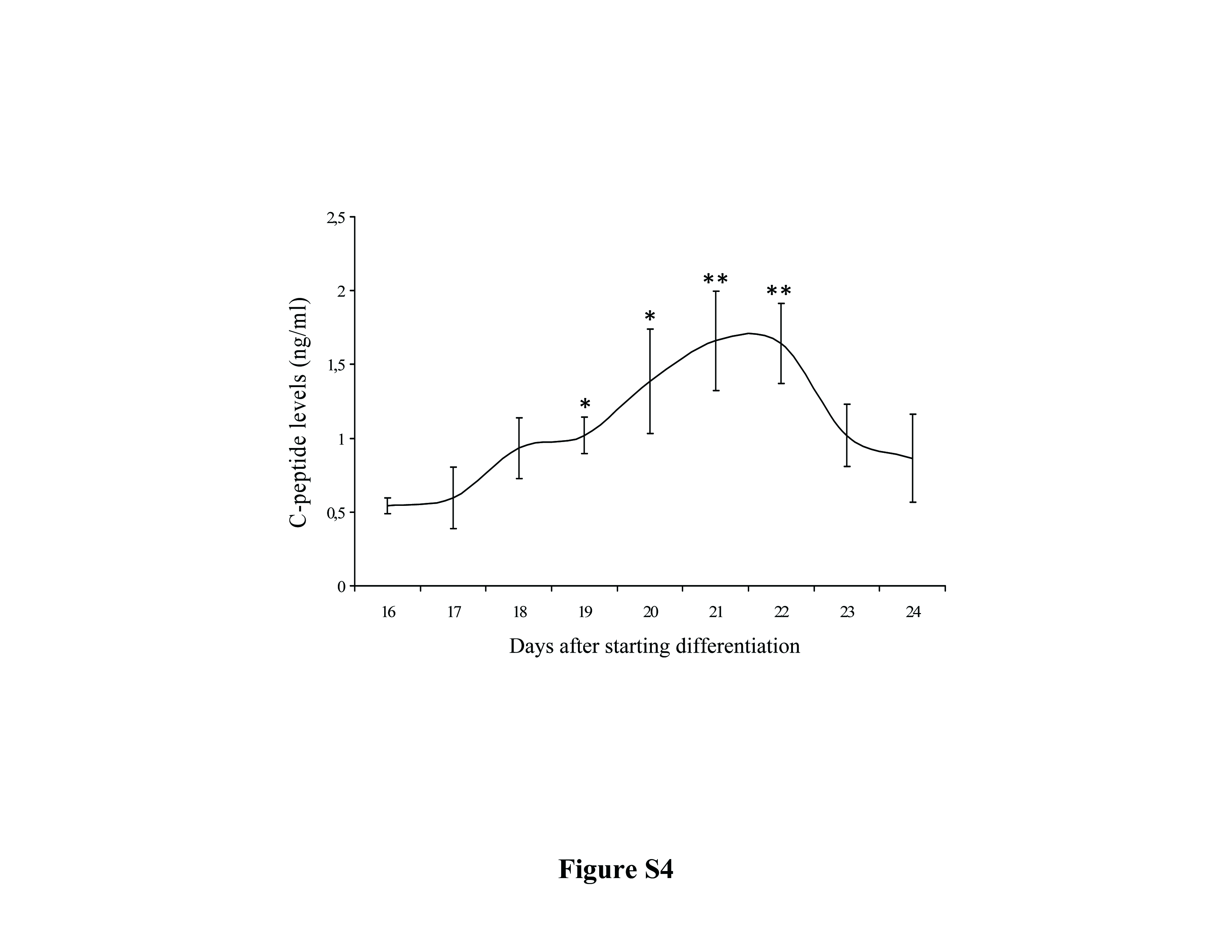

Supplement: Figure S4 — C-peptide release by rMAPC progeny during the course of differentiation. C-peptide secretion after one hour incubation with 20 mM glucose during the last 9 days (forth step+3 days) of the differentiation protocol was analyzed using rat C-peptide specific ELISA kit. The results shown are the average of three independent experiments. SEM±, n = 3 experiments. * p<0.05, ** p<0.01. (TIF) [file pone.0063491.s004.tif]

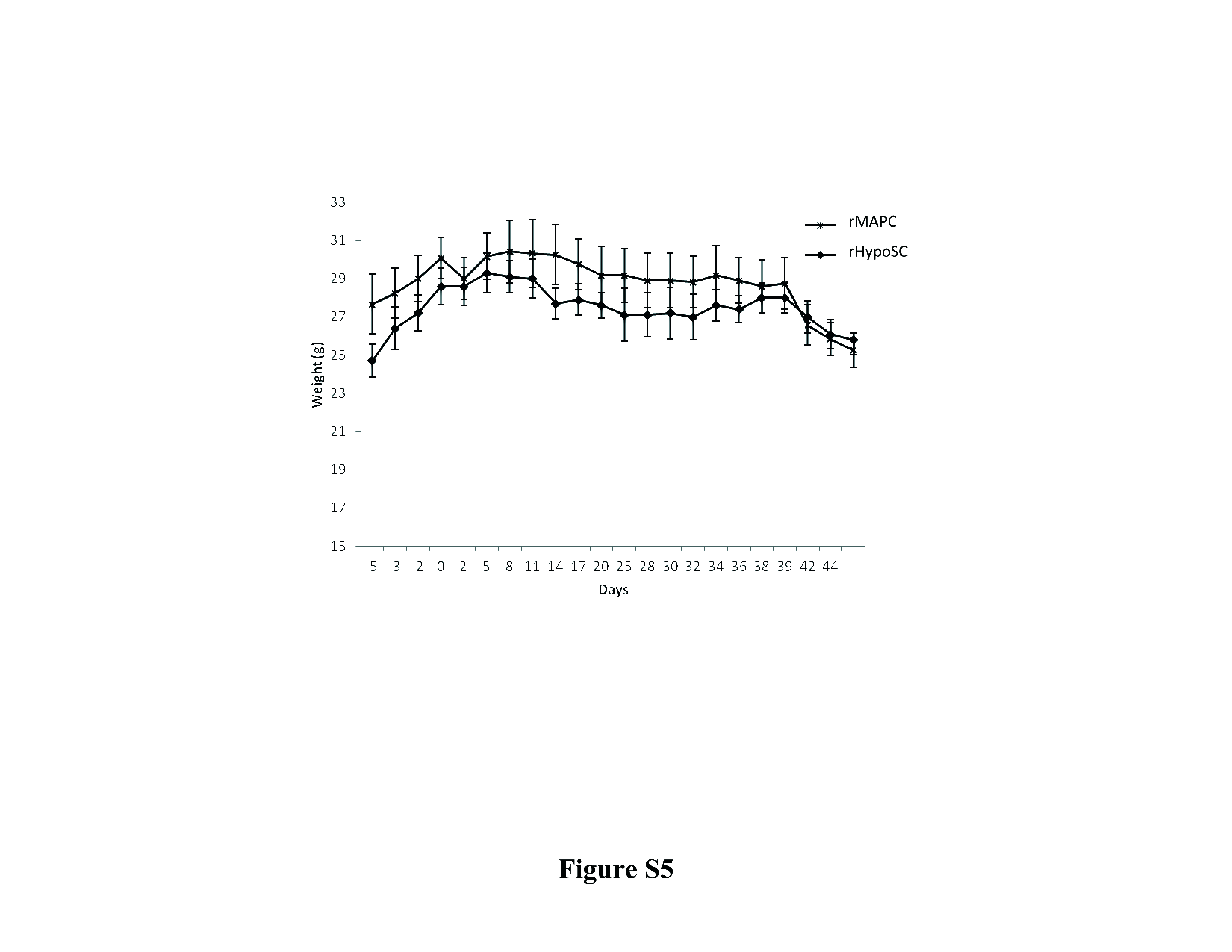

Supplement: Figure S5 — Analysis of body weight of the grafted animals. Body weight of mice transplanted with differentiated rMAPC (n = 6) and rHypoSC (n = 4) were monitored up to 45 days post transplantation. Data shown is mean ±SEM of the weight. (TIF) [file pone.0063491.s005.tif]

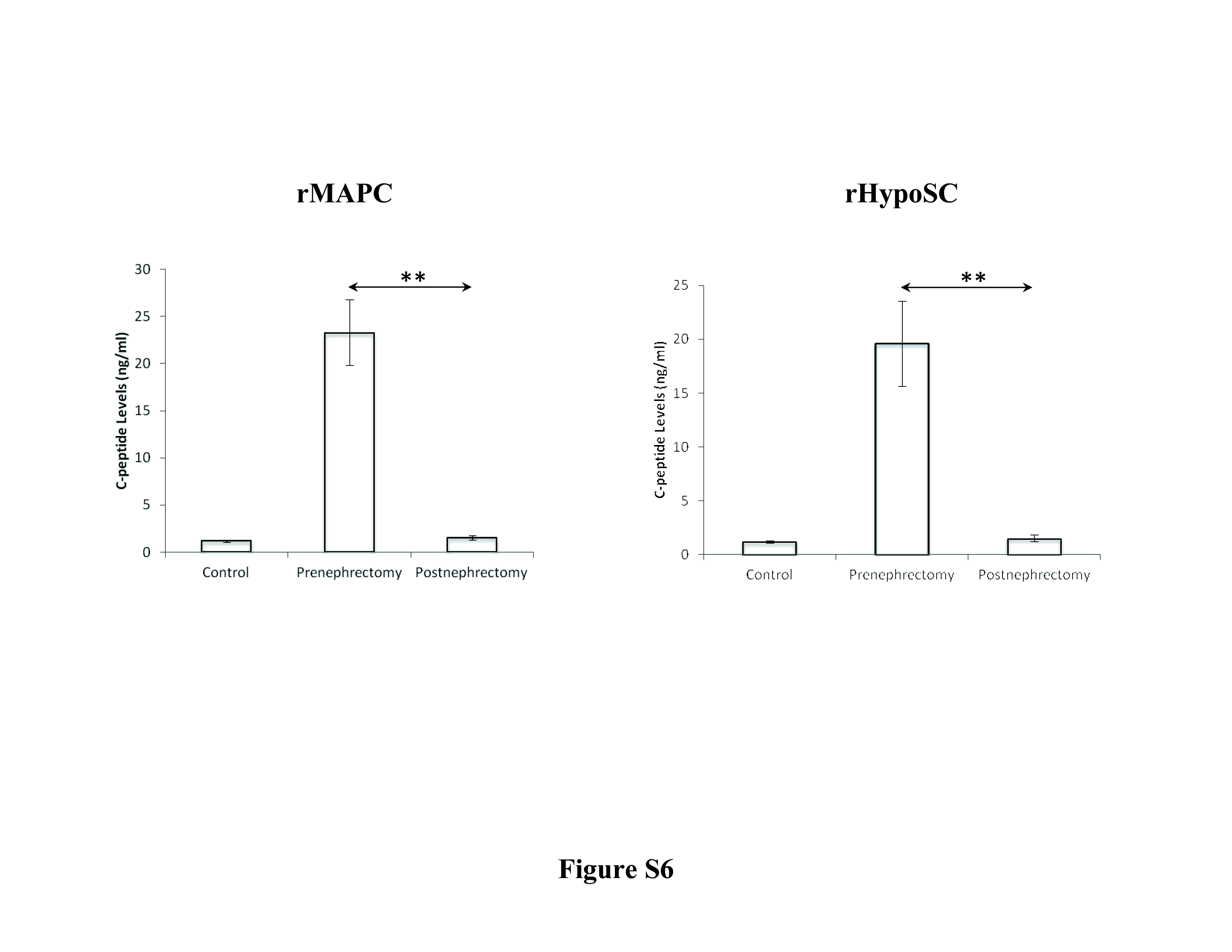

Supplement: Figure S6 — In vivo C-peptide analysis in mice transplanted with differentiated cells. Pre- and post-nephrectomized C-peptide release was measured in the serum of mice transplanted with differentiated (A) rMAPC (n = 6) and (B) rHypoSC (n = 4). Data shown is mean±SEM. ** p<0.01. (TIF) [file pone.0063491.s006.tif]

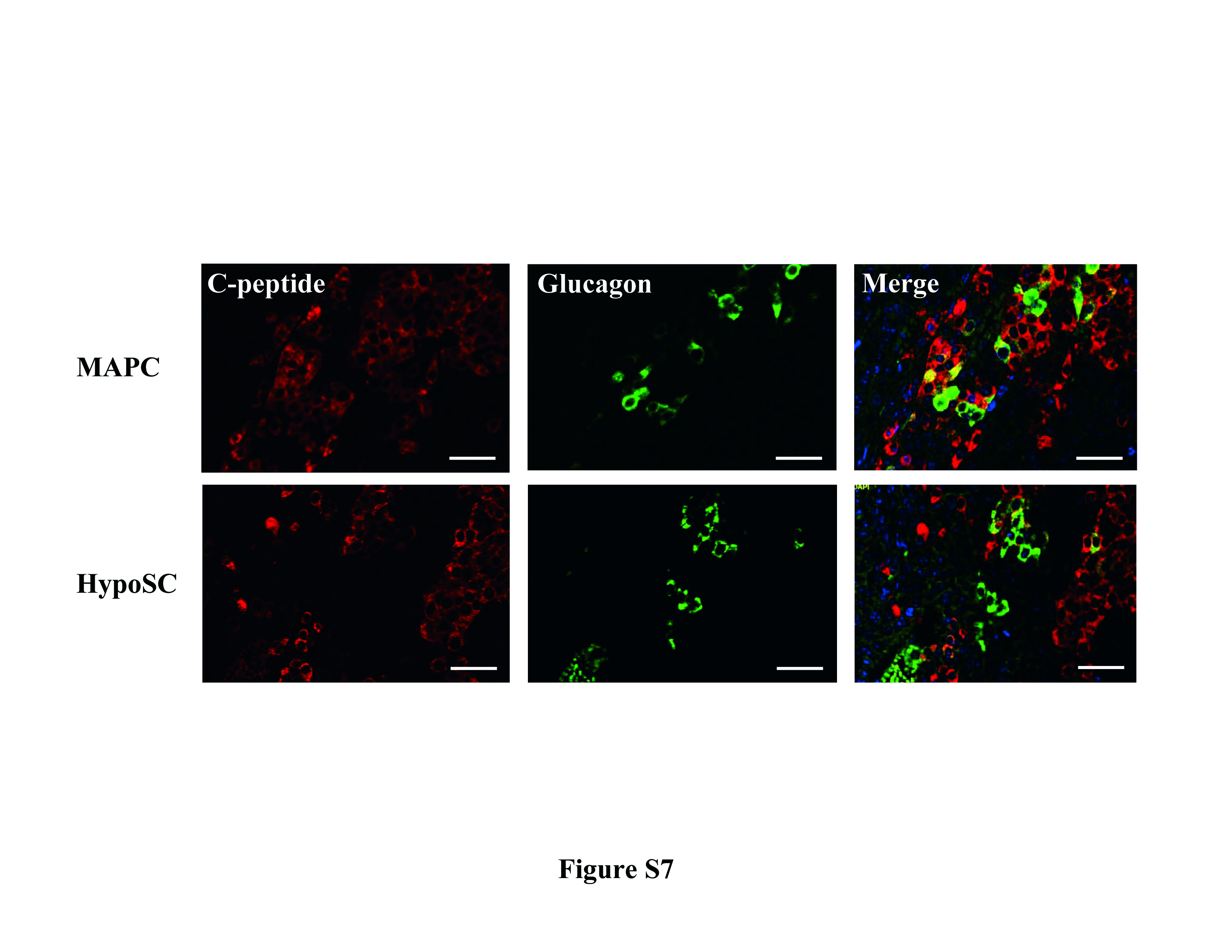

Supplement: Figure S7 — Immunohistological analysis of the graft. In some areas of the graft, colocalization of C-peptide and glucagon in both rMAPC and rHypoSC progeny was detected, indicating persistence of some immature hormone expressing cells. Scale bar = 50 µM. (TIF) [file pone.0063491.s007.tif]

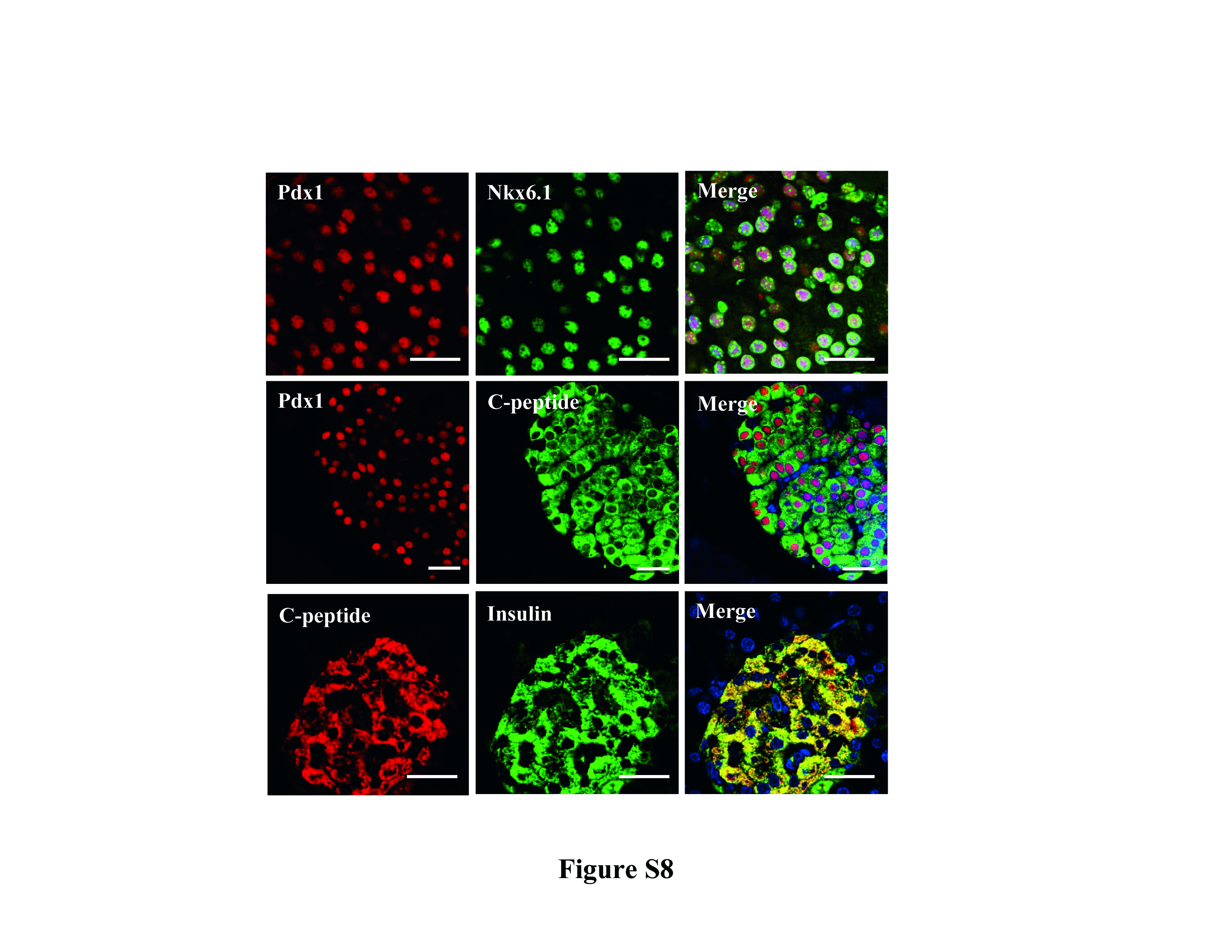

Supplement: Figure S8 — Immunohistological analysis of normal rat pancreas. As a positive control for the antibodies used for staining, normal rat pancreas were stained for islet hormones C-peptide and insulin and for transcription factors PDX1 and NKX6.1 along with nuclear specific staining using DAPI. Scale bar = 50 µM. (TIF) [file pone.0063491.s008.tif]
