# Supplementary material for: Reversal of Hyperglycemia by Insulin-Secreting Rat Bone Marrow- and Blastocyst-Derived Hypoblast Stem Cell-Like Cells
Source: PLoS One. 2013 May 9;8(5):e63491. doi: 10.1371/journal.pone.0063491 (PMC3650069; doi:10.1371/journal.pone.0063491)
Supplement: Table S1 — Quantitative PCR analysis of genes expressed during the course of differentiation of rMAPC1 to β-cell like cells (n = 3 experiments, ±SD). (DOCX) [file pone.0063491.s009.docx]

**Table S1:** Quantitative PCR analysis of genes expressed during the course of differentiation of rMAPC1 to β-cell like cells (n=3 experiments, ±SD)

|  | D0 | D3 | D6 | D9 | D15 | D18 | D21_2D | D21_3D | β−cells |
| --- | --- | --- | --- | --- | --- | --- | --- | --- | --- |
| *Oct4* | 4.8±0.12 | 6.9±0.24 | 9.8±1.1 | 10.3±1.22 | 11.1±0.32 | 12.8±2.22 | 11.9±1.28 | 10.7±0.34 | ND |
| *Mixl1* | 22.5±0.61 | 13.6±0.54 | 14.0±1.11 | 11.0±0.48 | 14.4±0.26 | 17.4±0.34 | 17.9±0.32 | 16.4±0.66 | ND |
| *Eomes* | 10.6±1.42 | 9.4±0.21 | 8.0±0.74 | 6.1±0.15 | 8.9±0.56 | 10.3±0.16 | 10.5±0.18 | 12.5±1.08 | ND |
| *Gsc* | 14.3±1.24 | 11.7±1.46 | 7.6±0.67 | 9.7±0.27 | 12.2±0.75 | 12.9±0.21 | 13.1±0.78 | 13.6±0.92 | ND |
| *CxcR4* | 16.3±1.36 | 11.7±1.24 | 7.4±0.98 | 10.2±1.75 | 11.3±2.62 | 13.4±2.31 | 10.2±1.96 | 11.2±2.13 | ND |
| *FoxA2* | 8.7±0.75 | 4.7±0.37 | 5.1±0.44 | 5.3±1.15 | 5.0±0.68 | 4.9±0.66 | 3.1±0.68 | 4.1±0.45 | ND |
| *Sox7* | 7.1±0.29 | 8.2±1.12 | 8.7±1.23 | 7.9±0.82 | 9.1±1.32 | 9.3±2.02 | 9.8±1.92 | 10.1±1.26 | ND |
| *Sox17* | 2.1±0.12 | 2.8±0.22 | 3.3±0.43 | 3.9±0.82 | 5.6±0.42 | 6.9±1.02 | 7.9±1.12 | 7.1±0.86 | ND |
| *Hnf1α* | 22.1±0.32 | 12.1±0.12 | ND | 14.2±1.12 | 11.4±0.62 | 10.4±1.02 | 5.4±0.82 | 8.5±1.32 | ND |
| *Hnf1β* | 5.1±0.22 | 4.8±1.0 | ND | 4.6±0.33 | 5.4±0.44 | 4.5±0.36 | 5.3±0.17 | 7.0±1.0 | ND |
| *Hnf4α* | 12.9±0.45 | 11.6±3.58 | ND | 7.4±0.94 | 8.5±0.62 | 8.2±1.26 | 8.3±0.77 | 11.7±1.98 | ND |
| *Hnf6* | 18.5±1.45 | 11.6±2.85 | ND | 8±1.35 | 9.5±2.75 | 7.2±1.28 | 5.8±1.12 | 8.4±0.62 | ND |
| *Pdx1* | 22.5±1.03 | 12.0±1.39 | ND | 13.9±0.56 | 11±1.99 | 9.3±1.49 | 9.4±0.32 | 5.7±0.66 | 2.7±0.32 |
| *Ngn3* | 23.0±1.66 | 21.9±0.96 | ND | 6.2±0.68 | 10.2±0.99 | 20.7±1.26 | 16.9±1.96 | 9.3±1.86 | ND |
| *NeuroD* | 22.8±0.48 | 17.6±1.87 | ND | 21.9±0.62 | 16.9±5.7 | 12.7±5.85 | 11.0±1.7 | 5.7±0.73 | ND |
| *Nkx2.2* | 15.3±0.13 | 13.9±2.23 | ND | 13.7±0.88 | 13.3±3.58 | 10.5±3.10 | 8.93±0.31 | 5.1±0.91 | ND |
| *Nkx6.1* | 15.2±0.38 | 15.5±1.28 | ND | 16.6±1.03 | 15.2±2.14 | 13.7±1.99 | 14.0±0.99 | 10.0±1.17 | 9.1±1.2 |
| *Pax4* | 18.5±0.83 | 16.7±1.28 | ND | 18.2±0.61 | 16.6±1.83 | 16.6±0.99 | 17.0±0.78 | 14.1±2.25 | ND |
| *Hlxb9* | 18.8±0.67 | 18.8±2.44 | ND | 13.0±0.64 | 14.3±1.48 | 13.4±1.90 | 12.9±0.60 | 8.6±0.52 | 12.1±1.2 |
| *Ins1* | 22.6±1.16 | 16.7±2.10 | ND | 20.8±0.77 | 14.4±6.38 | 9.8±7.04 | 6.08±0.95 | 2.0±0.57 | -6.3.±0.6 |
| *Ins2* | 21.1±0.77 | 16.4±2.62 | ND | 18.3±1.32 | 15.7±5.08 | 9.4±4.26 | 7.2±1.04 | 4.2±1.7 | -3.9.±0.7 |
| *Glp1R* | 21.4±0.89 | 16.4±2.62 | ND | 18.3±1.32 | 15.7±5.08 | 9.4±4.26 | 7.2±1.04 | 4.2±1.7 | 0.4±0.16 |
| *Isl1* | 21.1±0.44 | 18.5±1.23 | ND | 18.2±1.08 | 15.1±1.82 | 14.8±3.08 | 14.9±0.84 | 10.5±1.03 | 9.9±1.2 |
| *ABCC8* | 21.4±0.89 | 20.0±1.19 | ND | 20.9±0.55 | 19.9±2.16 | 17.2±2.46 | 15.8±1.87 | 12.5±2.96 | 11.8±1.2 |
| *Glut2* | 22.8±1.16 | 14.2±1.16 | ND | 7.6±1.16 | 8.0±1.16 | 7.3±0.98 | 8.2±0.53 | 9.1±0.78 | 3.1±0.86 |
| *Sst* | 22.0±0.66 | 20.0±0.98 | ND | 16.5±1.11 | 18.5±2.26 | 12.2±0.56 | 8.9±0.78 | 9.7±1.26 | ND |
| *Ghr* | 24.6±0.36 | 23.0±1.28 | ND | 22.5±1.13 | 19.4±2.14 | 18.1±0.35 | 9.1±1.57 | 12.7±2.02 | ND |
| *Amylase* | 24.8±1.23 | 21.2±2.13 | ND | 19.7±1.87 | 17.2±1.74 | 16.1±3.25 | 17.2±2.37 | 16.3±3.12 | ND |
| *Afp* | 21.0±0.64 | 7.5±6.17 | ND | 0.5±0.73 | 1.6±1.65 | 1.1±0.43 | 2.0±0.74 | 7.7±1.24 | ND |
| *Alb* | 22.2±1.15 | 15.8±2.94 | ND | 12.5±0.30 | 12.0±1.37 | 9.7±0.81 | 9.9±1.07 | 16.8±0.12 | ND |
| *GFAP* | 17.7±0.27 | 15.1±1.07 | ND | 15.1±0.53 | 15.6±0.09 | 15.8±0.46 | 16.3±0.83 | 16.3±1.46 | ND |
| *Flk1* | 20.0±0.96 | 14.9±1.16 | ND | 17.8±0.43 | 16.1±1.26 | 14.3±1.5 | 13.1±3.44 | 11.7±2.26 | ND |
| *vWF* | 21.3±0.55 | 16.1±2.9 | ND | 19.6±1.63 | 19.8±0.98 | 18.8±0.8 | 17.3±1.8 | 16.4±2.3 | ND |
| *VE Cadherin* | 14.6±0.45 | 16.4±3.0 | ND | 11.7±1.4 | 10.8±0.8 | 8.3±0.34 | 9.8±1.9 | 10.9±2.25 | ND |
