# Supplementary material for: Reversal of Hyperglycemia by Insulin-Secreting Rat Bone Marrow- and Blastocyst-Derived Hypoblast Stem Cell-Like Cells
Source: PLoS One. 2013 May 9;8(5):e63491. doi: 10.1371/journal.pone.0063491 (PMC3650069; doi:10.1371/journal.pone.0063491)
Supplement: Table S2 — Quantitative PCR analysis of genes expressed during the course of differentiation of CL19 to β-cell like cells (n = 3 experiments, ±SD). (DOCX) [file pone.0063491.s010.docx]

**Table S2:** Quantitative PCR analysis of genes expressed during the course of differentiation of CL19 to β-cell like cells (n=3 experiments, ±SD)

|  | D0 | D3 | D6 | D9 | D15 | D18 | D21_2D | D21_3D |
| --- | --- | --- | --- | --- | --- | --- | --- | --- |
| *Oct4* | 4.1±0.16 | 5.8±0.34 | ND | 8.3±1.62 | 10.3±1.54 | 11.6±2.35 | 10.9±2.38 | 11.4±1.54 |
| *Mixl1* | 23.4±1.16 | 11.2±1.44 | 11.0±0.91 | 9.1±0.88 | 11.3±1.1 | 15.5±1.2 | 16.9±1.62 | 15.4±1.10 |
| *Eomes* | 9.3±0.9 | 8.7±1.12 | 7.9±0.38 | 6.3±1.33 | 8.2±0.97 | 9.5±0.32 | 11.3±0.68 | 11.9±1.11 |
| *Gsc* | 15.8±1.22 | 11.3±1.2 | 6.2±0.9 | 7.9±0.83 | 10.3±1.12 | 14.3±0.99 | 15.8±1.12 | 13.3±1.22 |
| *CxcR4* | 14.1±1.55 | 8.2±1.56 | 6.3±1.36 | 7.6±1.34 | 9.2±1.23 | 10.6±1.22 | 9.1±2.69 | 8.1±1.17 |
| *Hnf3b* | 10.7±1.23 | 6.7±0.82 | ND | 5.1±1.28 | 4.0±0.96 | 4.3±0.75 | 3.9±0.59 | 6.2±0.66 |
| *Sox7* | 5.3±1.19 | 7.1±1.23 | ND | 8.3±1.62 | 10.2±2.42 | 9.5±2.62 | 10.6±1.99 | 11.1±1.33 |
| *Sox17* | 3.3±0.46 | 3.1±0.93 | ND | 4.6±0.91 | 5.9±1.33 | 7.2±1.01 | 8.2±1.36 | 7.9±1.88 |
| *Hnf1α* | 23.2±1.22 | 11.3±0.46 | ND | 10.4±0.89 | 9.3±0.63 | 8.2±1.12 | 6.5±0.99 | 6.6±1.11 |
| *Hnf1β* | 6.2±0.43 | 5.2±0.93 | ND | 4.4±0.26 | 4.9±0.32 | 5.2±0.36 | 5.8±0.41 | 5.3±0.66 |
| *Hnf4α* | 14.9±0.89 | 11.2±1.23 | ND | 6.8±0.86 | 6.1±0.98 | 7.9±1.12 | 7.3±1.23 | 8.9±1.11 |
| *Hnf6* | 19.2±1.16 | 10.2±1.23 | ND | 6.23±0.95 | 7.1±1.23 | 7.8±0.63 | 7.2±0.93 | 7.1±0.78 |
| *Pdx1* | 19.5±2.43 | 14.2±2.34 | ND | 15.1±1.86 | 10±2.32 | 7.2±2.46 | 8.2±1.33 | 6.9±1.32 |
| *Ngn3* | 21.0±0.96 | 19.7±1.92 | ND | 8.1±0.72 | 8.9±1.29 | 14.2±1.16 | 16.2±2.23 | 11.2±1.12 |
| *Pax4* | 20.6±1.33 | 15.9±1.46 | ND | 16.3±0.14 | 12.4±1.44 | 13.8±0.44 | 15.2±0.63 | 13.2±1.16 |
| *NeuroD* | 20.1±1.23 | 16.2±1.16 | ND | 18.2±1.12 | 13.9±3.26 | 11.1±2.85 | 9.0±1.89 | 5.1±1.14 |
| *Nkx2.2* | 17.3±1.13 | 12.8±1.27 | ND | 11.4±1.28 | 10.8±2.14 | 8.7±1.92 | 7.2±0.98 | 4.9±0.64 |
| *Nkx6.1* | 17.3±0.99 | 16.2±1.18 | ND | 14.3±1.24 | 15.1±1.46 | 12.1±0.89 | 11.1±1.23 | 8.9±0.97 |
| *Hlxb9* | 16.2±1.78 | 15.9±2.78 | ND | 16.1±1.46 | 13.8±2.36 | 11.2±2.67 | 9.6±1.72 | 7.2±1.32 |
| *Ins1* | 22.0±0.99 | 21.0±1.23 | ND | 20.3±1.13 | 16.2±3.28 | 12.6±2.14 | 7.1±1.23 | 3.2±0.78 |
| *Ins2* | 22.4±0.87 | 20.6±3.16 | ND | 19.2±2.44 | 17.8±2.16 | 15.6±3.18 | 9.1±2.14 | 3.9±0.96 |
| *Glp1R* | 22.3±2.16 | 19.4±1.13 | ND | 16.2±1.12 | 17.2±2.16 | 11.2±1.89 | 9.1±1.36 | 7.3±2.6 |
| *Isl1* | 19.2±1.24 | 17.2±1.12 | ND | 19.1±1.15 | 14.3±2.12 | 13.6±1.13 | 11.2±0.86 | 8.3±1.29 |
| *ABCC8* | 12.8±1.16 | 12.2±0.9 | ND | 11.8±1.16 | 10.6±1.11 | 10.8±1.12 | 9.6±1.2 | 8.8±1.10 |
| *Glut2* | 20.2±0.96 | 9.1±0.77 | ND | 6.2±1.23 | 5.9±0.47 | 6.8±0.79 | 7.1±0.75 | 6.4±0.55 |
| *Sst* | 23.1±1.24 | 22.4±1.28 | ND | 13.6±1.33 | 12.5±1.43 | 10.1±1.34 | 11.3±1.26 | 8.3±0.98 |
| *Ghr* | 23.8±1.26 | 22.1±1.16 | ND | 22.7±1.47 | 20.2±2.47 | 19.1±1.27 | 11.2±2.34 | 10.6±1.12 |
| *Amylase* | 22.7±1.46 | 19.3±1.42 | ND | 16.8±2.37 | 17.2±1.66 | 18.1±1.35 | 15.1±1.29 | 17.8±1.13 |
| *Afp* | 22.3±1.14 | 10.6±2.37 | ND | 2.6±0.99 | 1.3±0.78 | 2.9±0.93 | 4.8±1.34 | 9.2±2.34 |
| *Alb* | 22.0±1.24 | 18.8±2.24 | ND | 14.3±1.34 | 11.1±1.68 | 10.7±1.78 | 12.7±2.33 | 13.6±1.23 |
| *GFAP* | 20.1±1.38 | 17.1±1.56 | ND | 16.3±2.42 | 13.8±1.59 | 15.3±2.16 | 16.8±1.87 | 14.9±2.76 |
| *Flk1* | 22.6±1.86 | 17.8±1.98 | ND | 16.1±1.46 | 16.9±1.78 | 13.2±1.59 | 14.6±0.98 | 13.9±1.89 |
| *vWF* | 22.3±1.05 | 19.2±1.44 | ND | 19.8±1.93 | 19.9±0.72 | 19.2±0.72 | 18.4±0.92 | 17.3±1.21 |
| *VE Cadherin* | 16.9±1.36 | 17.3±1.23 | ND | 13.6±1.86 | 7.8±1.6 | 6.2±2.13 | 7.1±1.3 | 6.9±2.09 |
