# Supplementary material for: Reversal of Hyperglycemia by Insulin-Secreting Rat Bone Marrow- and Blastocyst-Derived Hypoblast Stem Cell-Like Cells
Source: PLoS One. 2013 May 9;8(5):e63491. doi: 10.1371/journal.pone.0063491 (PMC3650069; doi:10.1371/journal.pone.0063491)
Supplement: Table S3 — Quantitative PCR analysis of genes expressed during the course of differentiation of HypoSC to β-cell like cells (n = 3 experiments (Fi-1 = 2n and WK8 = 1n) ±SD). (DOCX) [file pone.0063491.s011.docx]

**Table S3:** Quantitative PCR analysis of genes expressed during the course of differentiation of HypoSC to β-cell like cells (n=3 experiments (Fi-1= 2n and WK8=1n) ±SD)

|  | D0 | D3 | D6 | D9 | D15 | D18 | D21_2D | D21_3D |
| --- | --- | --- | --- | --- | --- | --- | --- | --- |
| *Oct4* | 5.8±0.73 | 8.2±0.37 | 9.0±0.5 | 10.8±0.71 | 13.1±0.32 | 11.3±0.5 | 12.5±0.9 | 10.1±0.11 |
| *Mixl1* | 23.5±0.88 | 14.2±0.76 | 11.6±0.91 | 9.2±0.88 | 13.1±0.96 | 17.1±023 | 18.5±0.92 | 19.8±0.96 |
| *Eomes* | 10.5±0.98 | 9.5±0.43 | 7.5±0.82 | 5.4±0.99 | 8.0±1.36 | 10.8±1.12 | 11.2±0.78 | 13.5±1.23 |
| *Gsc* | 15.4±1.66 | 12.0±1.38 | 6.6±0.97 | 10.8±1.37 | 11.7±0.35 | 13.4±1.43 | 14.1±1.28 | 14.7±1.23 |
| *CxcR4* | 21.3±1.87 | 16.7±1.42 | 9.4±1.38 | 7.2±1.25 | 8.3±2.13 | 9.1±2.44 | 13.6±1.85 | 14.9±2.98 |
| *FoxA2* | 8.5±0.33 | 5.8±0.2 | 6.3±0.5 | 6.8±1.0 | 7.0±0.46 | 6.7±0.2 | 5.9±3.12 | 8.6±4.2 |
| *Sox7* | 6.2±1.09 | 6.8±1.02 | 7.1±0.98 | 8.9±0.62 | 10.7±0.92 | 11.6±1.12 | 11.9±0.76 | 13.2±1.36 |
| *Sox17* | 3.9±0.8 | 3.1±0.06 | 2.4±0.53 | 4.0±0.64 | 6.0±0.85 | 7.2±0.38 | 8.7±0.45 | 8.0±1.11 |
| *Hnf1α* | 22.3±0.19 | 9.9±3.8 | ND | 9.9±4.12 | 7.6±1.4 | 8.8±2.5 | 6.5±1.2 | 9.0±0.6 |
| *Hnf1β* | 5.0±0.22 | 4.8±0.7 | ND | 7.0±1.33 | 7.4±1.11 | 6.8±1.8 | 7.6±1.19 | 9.1±0.6 |
| *Hnf4α* | 14±1.14 | 13.8±2.2 | ND | 10.9±1.7 | 9.5±1.03 | 10.5±0.76 | 13.3±0.77 | 14.5±1.6 |
| *Hnf6* | 14.3±3.8 | 14.0±0.85 | ND | 7.8±0.9 | 5.2±1.6 | 4.6±1.9 | 7.0±1.8 | 7.2±1.3 |
| *Pdx1* | 22.3±0.3 | 21.0±2.8 | ND | 18.1±0.8 | 11.8±2.4 | 11.2±2.9 | 7.3±2.2 | 3.9±1.2 |
| *Ngn3* | 24.1±1.66 | 19.4±0.96 | ND | 8.1±1.23 | 5.2±1.28 | 13.2±2.43 | 14.8±2.46 | 15.7±2.93 |
| *NeuroD* | 21.3±1.1 | 21.9±1.2 | ND | 22.5±2.29 | 13.5±2.1 | 12.7±2.0 | 10.7±0.8 | 4.2±0.7 |
| *Nkx2.2* | 15.3±0.13 | 13.9±2.23 | ND | 13.7±0.88 | 13.3±3.58 | 10.5±3.10 | 8.9±1.2 | 4.1±0.21 |
| *Nkx6.1* | 16.8±0.75 | 16.0±1.7 | ND | 16.6±0.83 | 13.9±1.13 | 12.6±2.39 | 12.0±2.09 | 7.9±1.7 |
| *Pax4* | 22.3±0.5 | 20.7±1.5 | ND | 17.9±1.19 | 14.4±0.59 | 15.4±0.61 | 17.6±0.57 | 17.9±3.15 |
| *Hlxb9* | 22.6±0.2 | 21.5±1.7 | ND | 19.1±2.0 | 17.1±2.08 | 14.8±1.60 | 14.3±2.0 | 8.1±1.0 |
| *Ins1* | 22.0±0.8 | 22.2±0.9 | ND | 21.8±1.7 | 13.7±1.78 | 9.5±2.16 | 7.8±1.6 | 1.6±1.2 |
| *Ins2* | 21.8±1.2 | 22.0±0.96 | ND | 20.1±1.13 | 19.7±1.9 | 15.2±2.0 | 7.0±0.85 | 2.4±0.5 |
| *Glp1R* | 22.6±0.2 | 22.5±0.72 | ND | 18.4±0.89 | 11.4±1.5 | 10.1±1.16 | 11.2±1.13 | 8.2±1.7 |
| *Isl1* | 22.6±0.2 | 22.7±0.9 | ND | 19.8±1.2 | 14.2±1.19 | 13.8±0.45 | 12.9±1.3 | 11.2±1.7 |
| *ABCC8* | 22.6±0.2 | 22.7±0.9 | ND | 23.5±0.59 | 19.1±1.2 | 17.1±1.18 | 14.1±0.6 | 9.3±1.12 |
| *Glut2* | 22.2±0.7 | 13.2±3.7 | ND | 9.4±0.6 | 7.4±0.36 | 6.5±1.0 | 6.7±1.6 | 5.7±1.18 |
| *Sst* | 22.5±0.5 | 23.4±0.4 | ND | 22.7±1.0 | 17.8±0.9 | 14.8±1.3 | 13.0±1.0 | 12.5±1.06 |
| *Ghr* | 22.5±0.4 | 23.4±0.3 | ND | 23.2±0.5 | 15.9±0.35 | 13.0±0.8 | 11.4±0.12 | 8.4±0.15 |
| *Amylase* | 21.2±1.16 | 19.4±1.26 | ND | 17.7±1.23 | 19.1±1.17 | 20.1±1.17 | 19.3±1.43 | 21.3±1.34 |
| *Afp* | 23.0±1.44 | 10.4±2.37 | ND | 2.4±1.23 | 4.7±0.78 | 3.9±1.33 | 5.2±1.14 | 9.2±1.44 |
| *Alb* | 24.2±1.33 | 22.1±1.34 | ND | 18.5±1.23 | 16.1±1.14 | 13.4±1.21 | 14.3±1.2 | 17.1±1.42 |
| *GFAP* | 20.2±1.37 | 19.2±1.17 | ND | 18.3±1.72 | 19.4±1.19 | 17.8±1.26 | 16.9±1.23 | 19.5±1.44 |
| *Flk1* | 24.0±1.26 | 13.2±1.13 | ND | 12.2±1.23 | 11.1±1.16 | 12.1±1.6 | 11.2±1.24 | 10.2±1.36 |
| *vWF* | 22.4±0.65 | 19.2±1.23 | ND | 19.9±1.24 | 16.8±1.23 | 14.5±2.24 | 16.2±1.7 | 19.2±1.4 |
| *VE Cadherin* | 17.8±1.48 | 14.2±1.98 | ND | 9.2±1.55 | 9.8±1.26 | 10.8±1.39 | 7.8±1.69 | 11.3±2.16 |
