# Supplementary material for: Reversal of Hyperglycemia by Insulin-Secreting Rat Bone Marrow- and Blastocyst-Derived Hypoblast Stem Cell-Like Cells
Source: PLoS One. 2013 May 9;8(5):e63491. doi: 10.1371/journal.pone.0063491 (PMC3650069; doi:10.1371/journal.pone.0063491)
Supplement: Table S4 — Primer sequences used for the transcript detection by RT-qPCR. (DOCX) [file pone.0063491.s012.docx]

**Table S4:** Primer sequences used for the transcript detection by RT-qPCR.

| **Gene** | **Sequence** |
| --- | --- |
| *Oct4*  *Gsc*  *Eomes*  *Cxcr4*  *FoxA2*  *Sox7*  *Sox17*  *Hnf1β*  *Hnf1α*  *Hnf4a*  *Hnf6*  *Pdx1*  *Ngn3*  *Nkx6.1*  *NeuroD*  *Nkx2.2*  *Pax4*  *Ins1*  *Ins2*  *Isl1*  *Glut2*  *Sst*  *Ghr*  *Afp*  *Alb*  *Flk1*  *vwf*  *Vecad*  *Mixl1*  *Amy*  *Abcc8*  *Gfap* | Forward: 5’ GATGGCGTACTGTGGGCCC 3’  Reverse: 5’ TGGGACTCCTCCGGGTTTTG 3’  Forward: 5’ GAGAACCTCTTCCAGGAGAC 3’  Reverse: 5’ TTCTTAAACCAGACCTCCACC 3’  Forward: 5’ GAGGAGCAGTCGGATTTGAG 3’  Reverse: 5’ CCACTTCCACGAACACATTG 3’  Forward: 5’ GCAGGTAGCAGTGACCCTCT 3’  Reverse: 5’ CCAGGATTACCAACCCATTG 3’  Forward: 5’ GGAAACATTGGGGGAACTTT 3’  Reverse: 5’ GTGTGGCCCAGCTATTTAGG 3’  Forward: 5’ CAAGGATGAGAGGAAACGTC 3’  Reverse: 5’ CTCTGCCTCATCCACATAGG 3’    Forward: 5’ TGAGCAAGATGCTAGGCAAA 3’  Reverse: 5’ TAGTTGGGATGGTCCTGCAT 3’  Forward: 5’ GACACTCCTCCCATCCTCAA 3’  Reverse: 5’ ACATCAACCACCTCCCTCTG 3’  Forward: 5’ CAGCCACAACCATTCACATC 3’  Reverse: 5’ GCCATCTGGGTGGAGATAAA 3’  Forward: 5’ AAATGTGCAGGTGTTGACCA 3’  Reverse: 5’ CACGCTCCTCCTGAAGAATC 3’  Forward: 5’ CTGTGAAACTCCCCCAGGTA 3’  Reverse: 5’ TCATCCCGCATAAGTGTGAA 3’  Forward: 5’ TCTGCCTCTGGGACTCTTTC 3’  Reverse: 5’ GGGACCGCTCAAGTTTGTAA 3’  Forward: 5’ AACTGAGCACTTCGTGGTCCGA 3’  Reverse: 5’ CCAGACGCAATTTACTCCAGGC 3’  Forward: 5’ ACACCAGACCCACATTCTCC 3’  Reverse: 5’ GACCTGACTCTCCGTCATCC 3’  Forward: 5’ CCCAAAGCAAACAACCACTT 3’  Reverse: 5’ GTACCCCATCCTCCTGGAAT 3’  Forward: 5’ CAGCAGCGACAACCCCTAC 3’  Reverse: 5’ AAGAGCACTCGGCGCTTCC 3’  Forward: 5’ AGGACAAGGCTCCCAGTGTA 3’  Reverse: 5’ TAGGAAGAGCTGGAGCCAAA 3’  Forward: 5’ CACCTTTGTGGTCCTCACCT 3’  Reverse: 5’ GACGGGACTTGGGTGTGTAG 3’  Forward: 5’ GAAGTGGAGGACCCACAAGT 3’  Reverse: 5’ CAGTGCCAAGGTCTGAAGGT 3’  Forward: 5’ GGGACGGGAAAACCTACTGT 3’  Reverse: 5’ CACGAAGTCGTTCTTGCTGA3’  Forward: 5’ ATCCACATTCGGAACAGGAC 3’  Reverse: 5’ CAAGGTTCCGGTGATCTTGT3’  Forward: 5’ GAGCCCAACCAGACAGAGAA 3’  Reverse: 5’ GAAGTTCTTGCAGCCAGCTT 3’  Forward: 5’ CCAGCAGAGAAAGGAATCCA 3’  Reverse: 5’ GCTCCTCCTCTGCCTCTTCT 3’  Forward: 5’ ACCTGACAGGGAAGATGGTG 3’  Reverse: 5’ GTTCACAGGGTTTGCCTCAT 3’  Forward: 5’ TCTGCACACTCCCAGACAAG 3’  Reverse: 5’ AGTCACCCATCACCGTCTTC 3’  Forward: 5’ CCAAGCTCAGCACACAAAAA 3’  Reverse: 5’ CCAACCACTCTGGGAACTGT 3’  Forward: 5’ CCCACCGGATGGCTAGGTATT 3’  Reverse: 5’ GAGGCGGATCTGTTTGAGGTT 3’  Forward: 5’ GGCCAACGAATTGGATTCTA 3’  Reverse: 5’ GTTTACTGGCACCACGTCCT 3’  Forward: 5’ CGCCTCCTCCAGTCATGCT 3’  Reverse: 5’ CGCCAGAGTGGGAAGTCATT 3’  Forward: 5’ AGGAACATGGTTGCCTTCAG 3’  Reverse: 5’ AGTGCTTGACAAAGCCCAGT 3’  Forward: 5’ TCTTCGTCCTCGTGTGTGAG 3’  Reverse: 5’ AAGGCCAGGGTCCAGTAGAT 3’  Forward: 5’ GGTGGAGAGGGACAATCTCA 3’  Reverse: 5’ ACACAGCCAGGTTGTTCTCC 3’ |
